# Experimental and MEDT Study of Sydnone–Alkyne Cycloaddition-Based Synthesis of 1,4-Disubstituted Pyrazoles and In Silico Investigation of Their Binding to HCV and HIV Proteins

**DOI:** 10.3390/molecules31081250

**Published:** 2026-04-09

**Authors:** Souad Zerbib, Mohammed Eddahmi, Marwa Alaqarbeh, Pierre-Edouard Bodet, Valérie Thiery, Ahmed Fatimi, Natália Cruz-Martins, Christian Bailly, Luis R. Domingo, Latifa Bouissane

**Affiliations:** 1 Laboratory of Molecular Chemistry, Materials and Catalysis, Faculty of Sciences and Technologies, Sultan Moulay Slimane University, BP 523, Beni-Mellal 23000, Morocco; souadzerbib@gmail.com; 2 Sustainable Processes, Advanced Materials and Computational Chemistry Team, Polydisciplinary Faculty of Beni Mellal, Sultan Moulay Slimane University, Mghila, P.O. Box 592, Beni Mellal 23000, Morocco; eddahmi.med@gmail.com; 3 Applied Science Research Center, Applied Science Private University, Amman 11931, Jordan; marwaqarbh@gmail.com; 4 Plateforme d’Analyse Haute Résolution des Biomolécules PAHRB, LIttoral, ENvironment and Societies (LIENSs), UMR CNRS 7266, Université de La Rochelle, 17042 La Rochelle, France; pierreedouard.bodet@univ-lr.fr; 5 LIttoral, ENvironment and Societies (LIENSs), UMR CNRS 7266, Université de La Rochelle, Rue Olympe de Gouges, 17042 La Rochelle, France; valerie.thiery@univ-lr.fr; 6 Chemical Science and Engineering Research Team, Department of Chemistry, Polydisciplinary Faculty of Beni-Mellal, Sultan Moulay Slimane University, Beni-Mellal 23000, Morocco; a.fatimi@usms.ma; 7 Life and Health Sciences Research Institute (ICVS), School of Medicine, University of Minho, P.O. Box 4710-057 Braga, Portugal; nataliamartins@med.uminho.pt; 8 Institute of Pharmaceutical Chemistry Albert Lespagnol (ICPAL), Faculty of Pharmacy, University of Lille, rue du Professeur Laguesse, BP-83, F-59006 Lille, France; 9 Independent Researcher, Avd. Tirso de Molina 20, 46015 Valencia, Spain

**Keywords:** MEDT, CuSAC, 1,4-benzodioxane, sydnone, 1,4-disubstituted pyrazole, cyclophilin A, gp120 binding, molecular docking

## Abstract

Six 1,4-disubstituted pyrazoles linked to a benzenesulfonamide and a benzodioxane unit have been synthesized through a copper(I)-catalyzed formal [3+2] cycloaddition (32CA) reaction of alkynes with 3-arylsydnones. The Cu-catalyzed sydnone–alkyne cycloaddition (CuSAC) procedure has been optimized to promote the formation of the pyrazole ring and to deliver in three steps the six target compounds **5a**–**f**, fully characterized by ^1^H/^13^C-NMR and mass spectrometry (EIMS). Ten solvent conditions were evaluated. The reaction proceeded most efficiently in the presence of copper(II) sulfate pentahydrate in aqueous *t*-butanol in the presence sodium acetate, to reach a yield of 96%. The mechanism of the Cu(I)-catalyzed reaction has been studied within the Molecular Electron Density Theory (MEDT). This rection is a domino process that consists in a Cu(I)-catalyzed formal [3+2] cycloaddition followed of an extrusion of CO_2_ yielding the final pyrazole. The capacity of heterocyclic compounds **5a**–**f** to interact with human cyclophilin A (Cyp A), which is a host cofactor for hepatitis C virus (HCV) and human immunodeficiency virus 1 (HIV-1), and with the HIV-1 protein gp120-CD4 was evaluated using molecular docking. Compounds **5a,b,d,f** showed a satisfactory protein binding capacity. The physicochemical and metabolic properties of the compounds were also evaluated in silico. These predictions provide important information to guide future design in this series of potential antiviral agents.

## 1. Introduction

**Figure 1:**
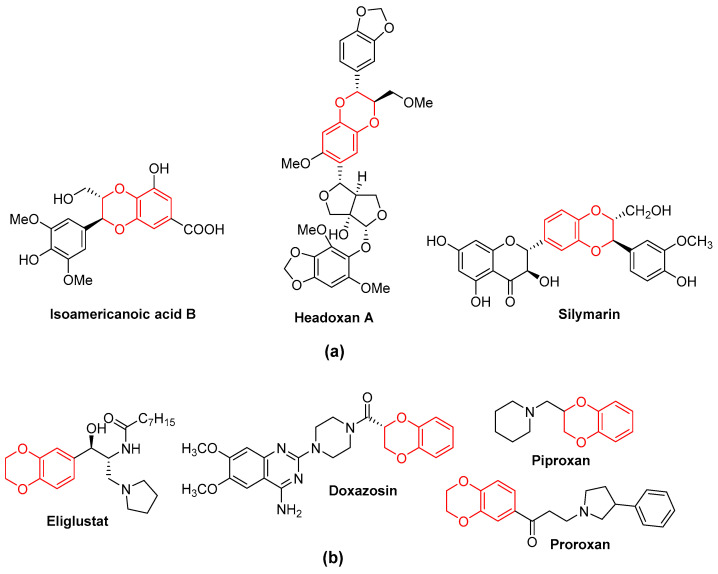
1,4-Benzodioxane unit (in red) in selected (**a**) natural products and (**b**) synthetic drugs.

Since its discovery in the late 1800s, the benzodioxane heterocycle has emerged as a successful unit for synthesizing biologically active compounds. Medicinal and bioorganic chemists have designed numerous benzodioxane-containing compounds endowed with biological and pharmacological activities [1]. Illustrative examples include isoamericanoic acid B (Figure 1a) extracted from the bark of the tree *Acer tegmentosum* Maxim, which represents a promising phytoestrogen targeting estrogen receptors α/β [2]. Headoxan A is a benzodioxane-containing natural product isolated from the East Asian perennial plant lopseed (*Phryma leptostachya* L.), which is a potent insecticidal lignan acting on axonal conduction and synaptic transmission in insects [3]. The flavonoid silymarin isolated from the seeds of the milk thistle plant *Silybum marianum* L. holds potent anti-inflammatory and antiviral properties, making it relevant for addressing both HCV- and HIV-induced inflammation [4]. Additionally, 1,4-benzodioxane derivatives exhibit a broad range of biological activities, including anti-inflammatory, antidiabetic, anticancer, and antifungal effects, and other pharmacological properties [5,6,7,8,9,10,11,12,13,14,15,16]. Furthermore, many important synthetic drugs incorporate this scaffold (Figure 1b), such as the glucosylceramide synthase inhibitor eliglustat [17], the drug doxazosin with smooth muscle relaxing activity [18], the anti-hypertensive proroxan (pyrroxane) [19], the antihistamine piperoxan [20], among others.

Today, a primary challenge is the development of efficient, cost-effective, and eco-friendly strategies to synthesize promising molecules. One approach to address this challenge is the development of cycloaddition reactions. Among others, the [3+2] cycloaddition (32CA) reaction is a chemical reaction between a three-atom-component (TAC) and a molecule with double or triple bond leading to the formation of five-membered heterocyclic compounds such as pyrazoles, pyrazolines, furans, triazoles, etc., while usually retaining the configuration of both the starting TAC and the ethylene [21,22].

Among TACs, sydnones (named after the city of Sydney, Australia) represent a well-known class of mesoionic compounds that play a significant role in heterocyclic chemistry and exhibit various biological activities [23,24,25,26,27,28]. They serve as intriguing partners for Cu(I)-catalyzed 32CA reactions with a variety of ethylenes, leading to the formation of 1,4-disubstituted pyrazoles. Their synthesis involves the nitrosation of *N*-amino acids followed by a cyclization of the resulting N-arylglycine in acetic anhydride [29,30,31,32,33]. Since Huisgen discovered the reaction in 1962, the reaction of sydnones with alkynes has been the subject of numerous studies [34]. This 32CA reaction proceeds via a thermal reaction with various ethylenes, yielding substituted pyrazoles, known for their diverse biological activities [29,35]. Initially, this reaction required high temperatures and prolonged reaction times, resulting in poor regioselectivity [36]. 

Through a screening approach, Taran and coworkers discovered that Cu(I) salts efficiently catalyze this reaction, yielding only 1,4-disubstituted pyrazoles under mild heating or at room temperature [37]. This reaction, termed CuSAC for Cu-catalyzed sydnone-alkyne 32CA reaction, has been demonstrated to be successful for bioconjugation with proteins. By exploring the CuSAC reaction, they reported the synthesis of over 100 fluorescent pyrazoles through the combination of 3-sydnone–coumarin with several alkynes [38]. Additionally, the synthesis of a broad spectrum of bromopyrazoles exhibiting excellent chemo and regioselectivities was achieved via the copper-catalyzed cycloaddition reaction of 4-bromosydnones with alkynes [39]. Wezeman and colleagues have recently optimized the synthesis of amino-pyrazoles from sydnone and *N*-substituted alkynes using Cu(OAc)_2_ and Cu(OTf)_2_ salts as catalysts [40]. However, the desired compounds were only obtained under CuSAC conditions. 

Many heterocycles with embedded pyrazole units have demonstrated a wide range of biological activities, including antioxidant and anti-inflammatory effects, leading to compounds with antimicrobial, antifungal, antiviral, antitumor, and antiparasitic properties [41,42,43,44,45]. In this context, we undertook the synthesis of pyrazoles linked to a 1,4-benzodioxane unit under CuSAC conditions. To this end, sydnones and alkylated sulfonamides were selected as model substrates to optimize the conditions for the target cyclization leading to the desired compounds. 

Here, the optimized synthesis and analytical characterization of six 1,4-disubstituted pyrazole derivatives incorporating a benzodioxane unit are reported. In parallel, a Molecular Electron Density Theory [46] (MEDT) study of the molecular mechanism of the Cu(I) catalyzed reaction yielding the final pyrazole is carried out, and a molecular docking study is performed to evaluate the potential of the synthesized compounds as ligands of cyclophilin A and gp120 proteins, which are involved in virus’s lifecycle. The cellular chaperone cyclophilin is a prolyl isomerase that binds to HIV-1 capsid protein A and is required for HIV-1 infectivity. It is a key therapeutic target for several viral diseases, including HCV infections. The HIV-1 envelope glycoprotein gp120, exposed on the surface of both HIV-1 virions and infected host cells, plays a pivotal role in viral entry by interacting with the CD4 receptor on host cells. Both cyclophilin A and gp120 proteins are considered as potential targets for drug design. For this reason, the binding of sydnone derivatives to both cyclophilin A and gp120 was investigated using molecular modeling. Such compounds may be of interest for preventing or treating viral infections and possibly other human diseases [47,48,49].

## 2. Results

### 2.1. Chemistry

**Scheme 1:**
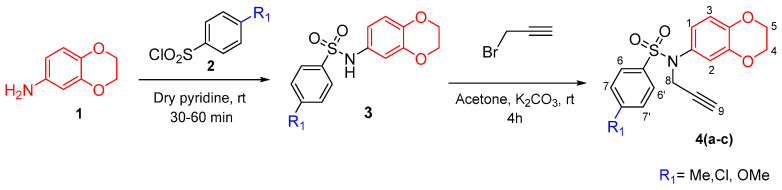
Synthesis of benzodioxan-containing sulfonamides **4a**–**c**.

*N*-(2,3-Dihydro-1,4-benzodioxin-6-yl)-4-arylsulfonamides **4a**–**c** were prepared in two steps. In the first step, a coupling reaction between *N*-2,3-dihydro-1,4-benzodioxine-6-amine **1** with 4-arylsulfonyl chlorides **2** in dry pyridine was carried out under constant stirring for 30–60 min at room temperature [42]. Afterwards, the *N*-alkylation of the sulfonamide **3** was conducted using propargyl bromide and potassium carbonate (K_2_CO_3_) in a polar aprotic solvent, acetone, leading to the desired benzodioxan-containing sulfonamides **4a**–**c** in excellent yields (93–95%) within 4 h, Scheme 1 [43].

**Scheme 2:**
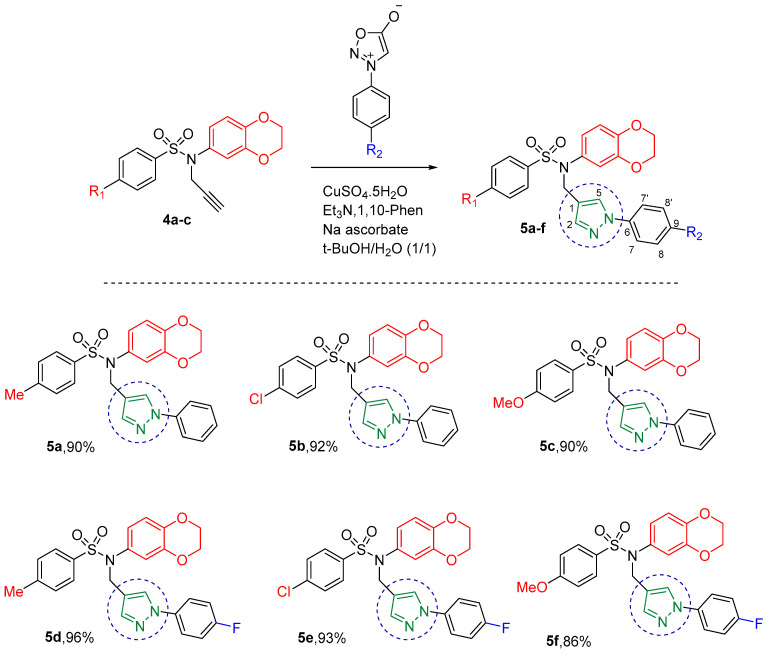
Synthesis of 1,4-disubstituted pyrazoles **5a**–**f** via Cu(I)-catalyzed 32CA reactions.

The structures of the compounds **4a**–**c**, Scheme 2, were unambiguously confirmed by NMR spectroscopy and mass spectrometry. The mass spectra of compounds **4a**–**c** exhibit a peak with an m/z value corresponding to the expected molecular ion [M+H]^+^. The 1D NMR spectra displayed signals in the range of δ 3.2–4.4 ppm, attributed to the methylene protons of the propargyl moiety. In the aromatic region, ^1^NMR spectra generally showed five signals for **4a** and **4c** and only four signals for **4b**, due to the resonances of the protons of sulphonyl aryl and benzene ring. These signals appear at ac. δ 7.51 and ac. δ 7.38 ppm for compounds **4a** and **4c**, respectively, whereas for compound **4b**, the analogous signal for H-7,7′ and H-6,6′ appeared as a multiplet in the range δ 7.72–7.58 ppm due to the resonance of the chlorine molecule at the para position of sulphonyl aryl. Furthermore, the analysis of the aliphatic region showed signals due to the resonance of the protons from the propynyl unit as a doublet at ac. δ 4.40 ppm and a singlet at ac. δ 3.2 ppm. Additionally, each ^13^C NMR spectra exhibited three signals at approximately δ 78.7, δ 76.7 ppm, and δ 40.9 ppm, respectively, attributed to the resonance of the carbons from the propynyl unit. The assignments of these signals were confirmed by DEPT 135 NMR spectra.

The *N*-(alkyl)-*N*-(2,3-dihydro-1,4-benzodioxin-6-yl)-4-arylbenzenesulfonamides **4a**–**c** were then reacted as terminal alkynes with sydnones to synthesize 1,4-disubstituted pyrazoles featuring a benzodioxine **5a–f**, Scheme 2.

Sequentially, sydnones were prepared through four steps. The first step involved the reaction of substituted anilines with ethyl chloroacetate in the presence of sodium acetate in ethanol to get anilino acetic ester. Subsequently, the latter was hydrolyzed, leading to its corresponding *N*-arylglycine before being subjected to nitrosation reaction and further cyclization with acetic anhydride to yield 3-arylsydnones. Sydnones are stable products at room temperature, and their synthesis does not require column chromatography, as the products of each synthetic step can be purified by simple recrystallization.

A systematic study of the 32CA reaction of ynamide as a terminal alkyne with sydnone was performed. We took into account most conventional approaches to the 32CA reaction of sydnones with alkynes [44]. Initially, we tried the classical procedure [45] for the synthesis of oxadiazole derivatives. However, fluoro-sydnone and compound **4a** were selected as a starting material to study the reaction conditions. Using xylene as a solvent, the reaction was followed for 72 h without yielding any cycloadduct (**entry 1**, Table 1). Additionally, the TLC analysis indicated a complete consumption of sulfonamide, suggesting a possible degradation due to the high temperature. Similar results were observed with toluene, as the reaction was heated at 120 °C (**entry 2**). Only a small amount of cycloadduct was formed when DMF was used as a solvent in the presence of copper(I) iodide as a catalytic source (**entry 3**). Substituting CuI with CuSO_4_.5H_2_O and using sodium ascorbate as a reducing agent in DMF/H_2_O mixed solvent led to the formation of the desired 1,4-disubstituted pyrazoles with only a percent yield of 30% (**entry 4**). This prompted us to explore milder conditions. Inspired by the CuSAC approach [36], we chose a mixed solvent, *t*-BuOH/H_2_O, preferably used in click chemistry, resulting in optimal yields at merely 60 °C. Initially, with 1.5 equivalents of *N*-alkyl sulfonamide, the reaction took 72 h without complete consumption of the dipolarophile. Conversely, by reducing the number of equivalents to 1eq, it required only 4 h for the reaction to yield the desired pyrazole, with excellent yield (**entry 8**). This can be explained by the in situ generation of Cu(I) from CuSO_4_ and sodium ascorbate, which activates the terminal alkyne via the formation of a copper acetylide. Furthermore, the t-BuOH/H_2_O solvent system increases solubility and stabilizes the transition state, allowing the reaction to proceed under milder conditions and resulting in higher yields. The solvent mixtures EtOH/H_2_O, DCM/H_2_O, and isopropanol/H_2_O showed no results (**entries 5**, **6,** and **7**). Similarly, no cycloadduct was formed using water as a solvent (**entry 10**). The variation of sydnone and *N*-alkyl sulfonamides was guided by the aim of introducing structural diversity and incorporating substituents that are frequently encountered in biologically relevant molecules. Fluorinated and chlorinated compounds were included in particular due to the prominent role of fluorine and chlorine in medicinal chemistry, where they are known to influence physicochemical properties and potential biological interactions.

The 1,4-disubstituted pyrazoles **5a**–**f** were fully identified based on ^1^D NMR spectroscopy and mass spectrometry. The mass spectra of compounds **5a**–**f** exhibit a peak with an *m*/*z* value corresponding to the expected molecular ion [M + H]+. The main features of the ^1^H-NMR spectra include the signals of the pyrazole hydrogen (H-5), appearing as a slightly unshielded singlet at approximately δ 8.29 ppm, and hydrogen (H-2), appearing as a singlet at around δ 7.49 ppm. The hydrogens of the *N*-phenyl ring are confirmed by two multiplets in the range of approximately δ 7.7 and δ 7.15 ppm. The signal of hydrogen (H-9) appears as a triplet at ca. δ 7.27 ppm with *J* = 7.4 Hz for compound **5a** and as a multiplet in the range δ 7.3 and δ 7.25 for compounds **5b** and **5c**. In addition to the presence of the pyrazole ring hydrogen signals (H-5) and (H-2) at ca. δ 8.28 and δ 4.63 ppm respectively, the structure of compounds **5d**–**f** is confirmed by the absence of the hydrogen (H-9) at ca. δ 7.27 ppm for compounds **5a**–**c**, which is replaced by a fluorine atom. All the other NMR signals are reliable with the structure, and some multiplicities are influenced by the 19F-1H heteronuclear spin–spin coupling. The ^13^C NMR spectra are also in good agreement with the structures of compounds **5a**–**f**. Each spectrum shows a signal at approximately δ 140.8, δ 126–127, and δ 116.3 ppm due to the resonance of the C-2, C-5, and C-1, respectively, of the pyrazole type ring. Signals from the *N*-aryl ring are present in the range of δ 127 to δ 116 ppm, as well as the C-9 signal, which appears to be the most unshielded at around δ 159 ppm for **5d–f** due to the presence of the fluorine atom.

### 2.2. MEDT Study of the Cu(I)-Catalyzed Formation of Pyrazole **8**

**Scheme 3:**
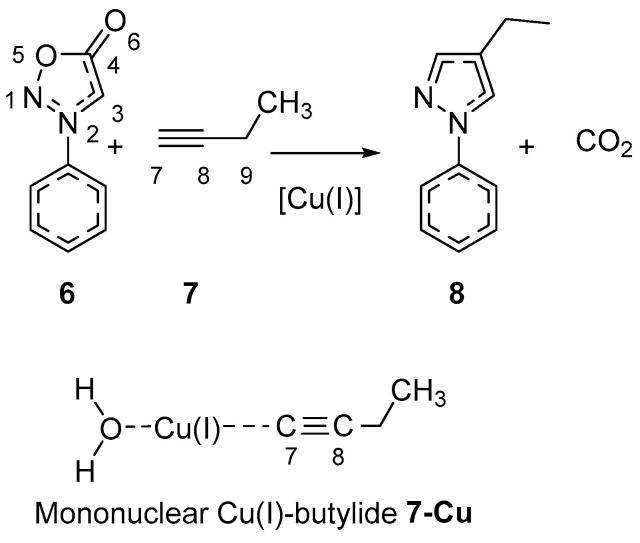
Reaction of oxadiazole **6** with butyne **7** yielding pyrazole **8**, in the absence and in the presence of Cu(I) as catalyst and mononuclear Cu(I)-butylide complex **7-Cu**.

To gain deeper insight into the molecular mechanism of the Cu(I) catalyzed reactions, an MEDT [46] study at the ωB97X-D/6-311G(d,p) computational level in solution of the domino reaction of oxadiazole **6** with butyne **7**, as a reduced model of the sulfonamides **4a**–**c**, in the absence and in the presence of Cu(I) as catalyst were studied (see Scheme 3). The sulfonamide frameworks of alkynes **4a**–**c**, which are attached to the tetrahedral C9 carbon, were replaced by a methyl group in butyne **7**. This significantly reduced the size of sulfonamides **4a**–**c**. In this MEDT study, the mononuclear Cu(I)-butylide complex **7-Cu**, in which the cation Cu(I) is coordinated to the butynyl anion and one water molecule, was used in the study of the Cu(I)-catalyzed reaction, Scheme 3 [50].

#### 2.2.1. ELF Topological Analysis of Oxadiazole **6**, Butyne **7**, and Mononuclear Cu(I)-Butylide **7-Cu**

**Figure 2:**
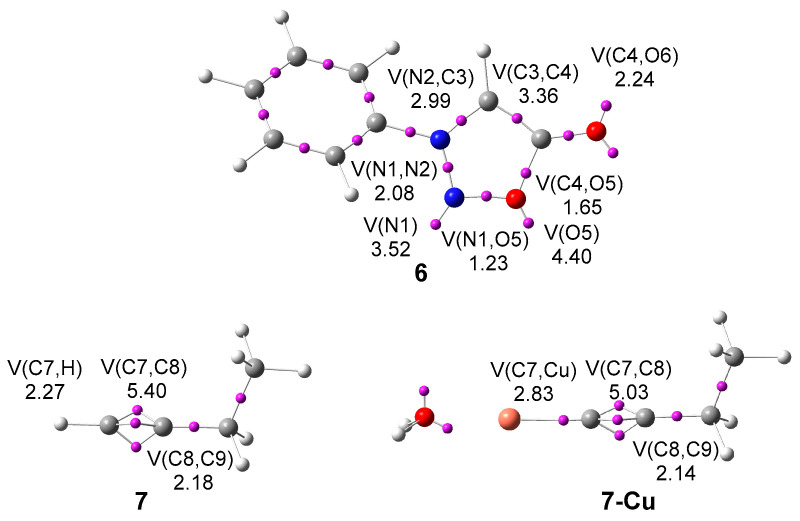
*ω*B97X-D/6-311G(d,p) ELF basin attractor positions, along with the more relevant valence basin populations of oxadiazole **6**, butyne **7**, and mononuclear Cu(I)-butylide **7-Cu**. Valence basin populations are given as the average number of electrons, e.

The electron topological analysis of the Electron Localization Function (ELF) [51] provides a quantitative description of the way electron density is organized in molecular systems, thus providing a direct connection between the electronic structure and chemical behavior. In the ELF framework, the notation proposed by Silvi and Savin [52] is employed: V(A,B) denotes a disynaptic basin associated with a shared electron pair between atoms A and B (i.e., a bonding region), whereas V(A) represents a monosynaptic basin corresponding to localized non-bonding or core/valence electron density on atom A. Using this formalism, an ELF topological analysis was carried out for oxadiazole **6**, butyne **7**, and mononuclear Cu(I)-butylide **7-Cu**. The positions of the ELF basin attractors and the more relevant valence basin populations of these species are presented in Figure 2.

The ELF of oxadiazole **6** shows the presence of one V(N1,N2) disynaptic basin integrating 2.08 e, one V(N2,C3) disynaptic basin integrating 2.99 e, one V(C3,C4) disynaptic basin integrating 3.36 e, one V(C4,O5) disynaptic basin integrating 1.65 e, one V(N1,O5) disynaptic basin integrating 1.23 e, and one V(C4,O6) disynaptic basin integrating 2.24 e. Note that the N1-N2-C3 framework of oxadiazole **6** will act as the TAC in the formal 32CA reaction of oxadiazole **6** with butyne **7**. The absence of a V(C3) monosynaptic basin, which characterizes a *pseudocritical* or carbenoid carbon, in oxadiazole **6** characterizes the N1-N2-C3 framework of this species as a zwitterionic TAC that participates in a *zw-type* 32CA reaction [53]. This behavior requires the involvement of electrophilic/nucleophilic species to favor the *zw-type* 32CA reaction.

The ELF of butyne **7** shows the presence of three V(C7,C8) disynaptic basins integrating a total of 5.40 e, one V(C8,C9) disynaptic basin integrating 2.18 e, and one V(C7,H) protonated basin integrating 2.27. On the other hand, mononuclear Cu(I)-butylide **7-Cu** shows the presence of three V(C7,C8) disynaptic basins integrating a total of 5.03 e, one V(C8,C9) disynaptic basin integrating 2.14 e, and one V(C7,Cu) disynaptic basin integrating 2.83 e. The formation of complex **7-Cu** reduces the electron density of the C7-C8 bonding region by 0.37 e as a consequence of the polarization of the electron density Cu-C7-C8 region towards the cation Cu(I).

#### 2.2.2. Analysis of the DFT-Based Reactivity Indices 

The study of DFT-based reactivity indices [54,55] provides valuable insights into reactivity within polar reactions [56]. The reactivity indices were calculated at the B3LYP/6-31G(d) level, which was originally employed to establish the standard electrophilicity and nucleophilicity scales [55]. The global reactivity indices, including electronic chemical potential μ, chemical hardness η, electrophilicity ω, and nucleophilicity *N*, for oxadiazole **6**, butyne **7**, and Cu(I)-butylide **7-Cu** are compiled in Table 2.

The electronic chemical potential *μ* [57] of butyne **7**, and mononuclear Cu(I)-butylide **7-Cu**, −2.73 and −3.03 eV, respectively, are above that of oxadiazole **6**, μ = −4.13 eV. Consequently, along a polar reaction the global electron density transfers [58] (GEDT) will take place from **7** or **7-Cu** towards **6**, in a reaction classified as reverse electron density flux [59] (REDF).

The electrophilicity ω [60] and nucleophilicity *N* [61] indices of oxadiazole **6**, 2.22 and 3.07 eV, respectively, classify this species as strongly electrophile and strongly nucleophile within the standard electrophilicity and nucleophilicity scales [55]. Thus, oxadiazole **6** can participate as an electrophile or nucleophile in polar reactions.

Butyne **7**, with ω = 0.44 and *N* = 2.10 eV, is classified as a marginal electrophile and a moderate nucleophile. Consequently, it is expected that butyne **7** will not participate in polar reactions. The formation of mononuclear Cu(I)-butylide **7-Cu** increases its electrophilicity to 1.12 eV and its nucleophilicity to 4.05 eV. Therefore, **7-Cu** is classified as a moderate electrophile and a strong nucleophile. Its nucleophilicity *N* index, which is higher than 4.0 eV, classifies this species as supernucleophile [56]. Consequently, it is expected that mononuclear Cu(I)-butylide **7-Cu** participates as a strong nucleophile towards the electrophilic oxadiazole **6** in a polar reaction.

The analysis of the DFT-based reactivity indices indicates that the *zw-type* 32CA reaction of oxadiazole **6** with butyne **7** will be non-polar and have a high activation energy, whereas the reaction involving the supernucleophilic Cu(I)-butylide **7-Cu** will be polar and have a low activation energy.

#### 2.2.3. Study of the Domino Reaction of Oxadiazole **6** with Butyne **7**

**Scheme 4:**
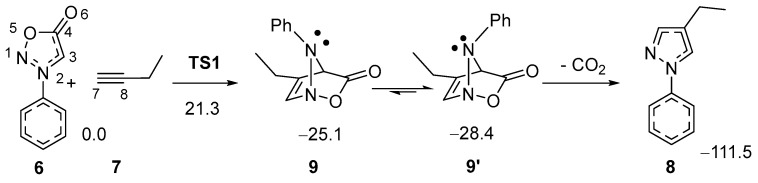
Domino reaction between oxadiazole **6** and butyne **7** yielding pyrazole **8**. The *ω*B97X-D/6-311G(d,p) relative energies in solvent are given in kcal·mol^−1^.

Next, the potential energy surface (PES) of the reaction of oxadiazole **6** with butyne **7** yielding pyrazole **8** was studied. This reaction is a domino process that comprises two consecutive reactions, Scheme 4: (i) a formal 32CA reaction of the N1-N2-C3 framework of oxadiazole **6** participating as the TAC with butyne **7** to yield the heterobicyclic compound **9**; and (ii) a retro-32CA reaction involving the extrusion of CO_2_ at compound **9′** yielding the final pyrazole **8**.

The first formal 32CA reaction of oxadiazole **6** with butyne **7** yielding heterobicyclic compound **9** takes place via a one-step mechanism via **TS1**, which is found to be 21.3 kcal·mol^−1^ about the separated reagents. The formation of heterobicyclic compound **9** is exothermic by −25.1 kcal·mol^−1^. The heterobicyclic compound **9** is stable, but by an inversion of the pyramidal N2 nitrogen equilibrate with its conformer **9′** that is 3.3 kcal·mol^−1^ more stable than **9**. Finally, the heterobicyclic compound **9′** spontaneously releases CO_2_ with no appreciable barrier yielding the final pyrazole **8**. The global domino reaction is strongly exothermic by −111.5 kcal·mol^−1^.

**Figure 3:**
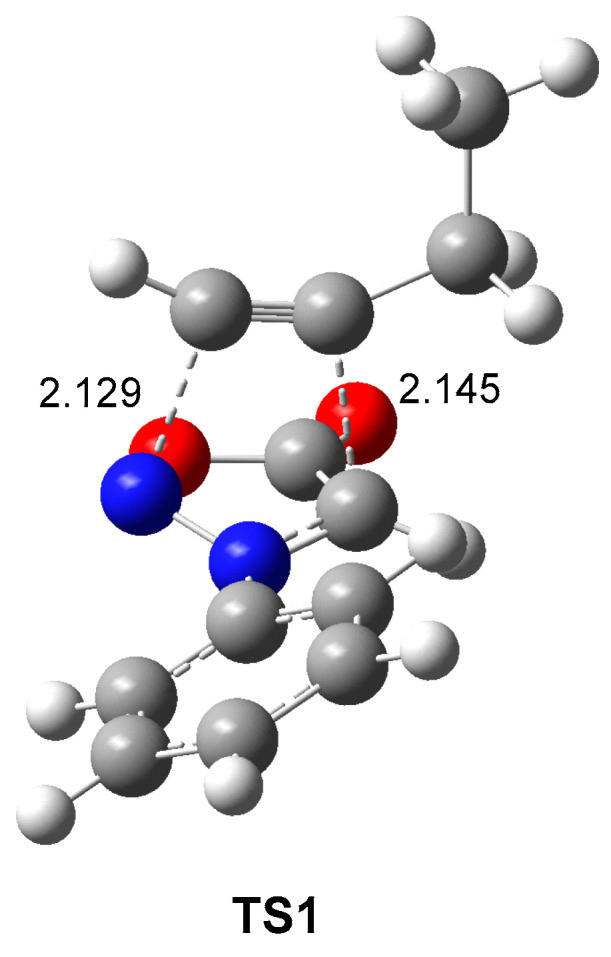
*ω*B97X-D/6-311G(d,p) optimized geometry of **TS1**. The distances are given in Angstroms.

The optimized geometry of **TS1** in solvent is given in Figure 3. At **TS1,** the C-C and C-N distances are 2.145 and 2.129 Å, respectively. These distances, which exceed 2.0 Å, suggest that neither C-C nor C-N single bonds are formed through this TS [58].

Finally, analysis of the GEDT [58] at the TS allows for evaluating the polar nature of the 32CA reaction. GEDT values lower than 0.05 e are characteristic of non-polar processes, whereas values exceeding 0.20 e indicate highly polar reactions. The GEDT at **TS1** is 0.02 e. This very low value, which is a consequence of the very low nucleophilic character of butyne **7**, see Table 1, characterizes the non-polar character of the formal 32CA reaction, which is classified as null electron density flux (NEDF) [59].

#### 2.2.4. Study of the Domino Reaction of Oxadiazole **6** with Mononuclear Cu(I)-Butylide **7-Cu**

**Scheme 5:**
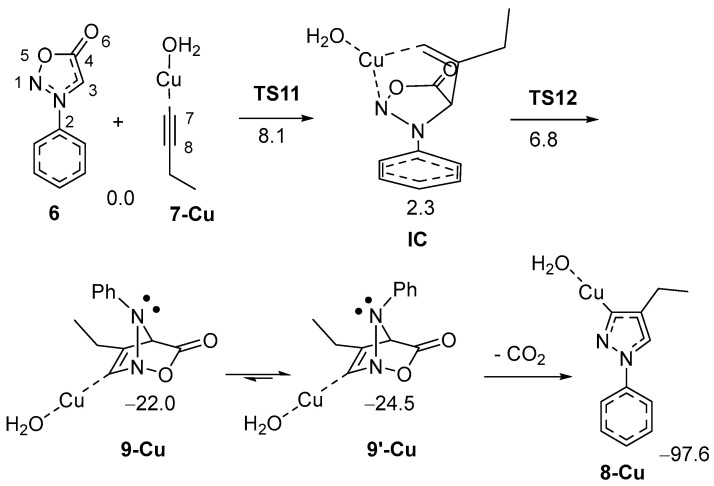
Domino reaction of oxadiazole **6** with mononuclear Cu(I)-butylide **7-Cu**. The *ω*B97X-D/6-311G(d,p) relative energies in solvent are given in kcal·mol^−1^.

The reaction of oxadiazole **6** with mononuclear Cu(I)-butylide **7-Cu** is a domino process that comprises two consecutive reactions, as shown in Scheme 5: (i) a formal 32CA reaction of the N1-N2-C3 framework of oxadiazole **6** acting as the TAC with mononuclear Cu(I)-butylide **7-Cu** to yield the heterobicyclic compound **9-Cu**; and (ii) a retro-32CA reaction at **9′-Cu** involving the extrusion of CO_2_ yielding the final pyrazole **8-Cu**. Analysis of the PES associated with the first Cu(I)-catalyzed formal 32CA reaction indicates that it takes place via a two-step mechanism with formation of an intermediate complex **IC**, as shown in Scheme 5.

The formation of heterobicyclic compound **9-Cu** occurs in a two-step mechanism. The first step, associated with the nucleophilic attack of the C8 carbon of mononuclear Cu(I)-butylide **7-Cu** on the C3 carbon of oxadiazole **6** via **TS11**, has an activation energy of 8.1 kcal·mol^−1^; the formation of the intermediate complex **IC** is slightly endothermic by 2.3 kcal·mol^−1^. The second step associated with the formation of the N1-C7 single bond via **TS12** has an activation energy of 4.5 kcal·mol^−1^ from **IC**, and the formation of heterobicyclic compound **9-Cu** is exothermic by −22.0 kcal·mol^−1^. The heterobicyclic compound **9-Cu** is stable, but by an inversion of the pyramidal N2 nitrogen, it equilibrates with conformer **9′-Cu**, which is 2.5 kcal·mol^−1^ more stable than **9-Cu**. Finally, the heterobicyclic compound **9′-Cu** spontaneously releases CO_2_ with no appreciable barrier in a retro-32CA reaction yielding the final Cu-pyrazole complex **8-Cu**. The global domino reaction is strongly exothermic by −97.6 kcal·mol^−1^.

A comparison of the domino reaction of oxadiazole **6** with butyne **7** in the absence of Cu(I), as shown in Scheme 4, and in the presence of the Cu(I)-catalyst, as shown in Scheme 5, allows us to obtain three appealing conclusions: (i) while the formal 32CA reaction in the absence of Cu(I) takes place via a one-step mechanism, that in the presence of Cu(I)-catalyst takes place via a two-step mechanism; (ii) the thermodynamics of the two domino reactions in the absence and in the presence of Cu(I)-catalyst are very similar; while the first 32CA reactions are exothermic by ca. 20 kcal·mol^−1^, the overall domino processes are strongly exothermic by ca. 100 kcal·mol^−1^; and iii) while the non-catalyzed 32CA reaction presents an activation energy of 21.3 kcal·mol^−1^, that associated with the Cu(I)-catalyzed process has an activation energy that is 13.8 kcal·mol^−1^ lower than that of the non-catalyzed process.

**Figure 4:**
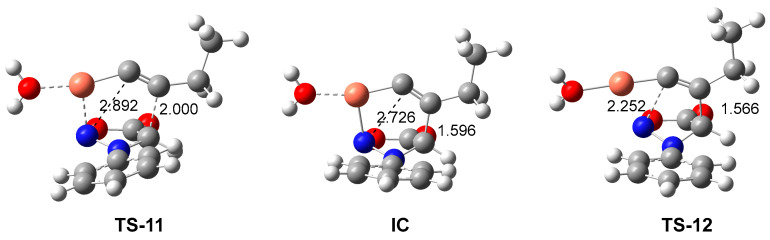
*ω*B97X-D/6-311G(d,p) optimized geometries of **TS11**, **IC**, and **TS12**. The distances are given in Angstroms.

The optimized geometries of **TS21**, **IC**, and **TS22** are given in Figure 4. At **TS11**, the distance between the interacting C3 and C7 carbons is 2.000 Å, while the distance between the N1 and the C8 atoms is 2.892 Å. At this TS, the Cu(I) cation is also coordinated to the N1 atom. At the intermediate complex **IC,** the C3-C7 distance is 1.597 Å, according to a C-C single bond, while the distance between the N1 and the C8 atoms remains at 2.726 Å. Finally, at **TS-12,** the C3-C7 bond length is 1.597 Å, while the distance between the interacting N1 and C9 atoms is reduced to 2.252 Å.

Finally, the GEDT [58] was computed at **TS11**. At this TS, the GEDT has a high value, 0.31 e, indicating the high polar character of this Cu(I)-catalyzed formal 32CA reaction, classified as REDF in complete agreement with the analysis of the DFT-based reactivity indices, which classify oxadiazole **6** as strongly electrophile and mononuclear Cu(I)-butylide **7-Cu** as supernucleophile, see Table 2. Recent MEDT studies have shown that the GEDT taking place at the polar TSs is responsible for the reduction in activation energies by a stabilization of the electrophilic ethylene [62,63].

In the Cu(I)-catalyzed formal 32CA reaction, the Cu(I) cation strongly stabilizes the alkynyl anion obtained by deprotonation of butyne **7**, rendering mononuclear Cu(I)-butylide **7-Cu** as supernucleophile and facilitating the GEDT to the strong electrophilic oxadiazole **6**. This phenomenon strongly stabilizes the electrophilic framework at the TS, reducing the activation energy of the non-polar 32CA reaction [62,63].

#### 2.2.5. ELF Topological Analysis of TSs and IC Involved in the Cu(I)-Catalyzed Formal 32CA

**Figure 5:**
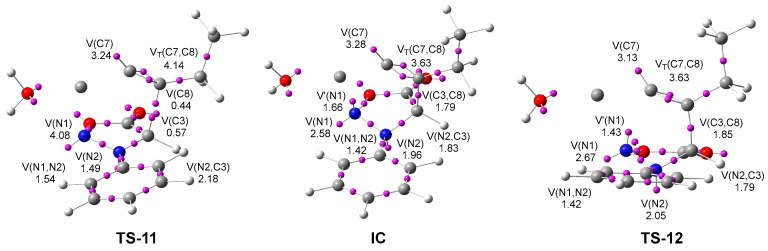
*ω*B97X-D/6-311G(d,p) ELF basin attractor positions, along with the most relevant valence basin populations of **TS11**, **IC**, and **TS12**. Valence basin populations are given as the average number of electrons, e.

Finally, an ELF topological analysis of **TS11**, **IC**, and **TS12** involved in the Cu(I)-catalyzed formal 32CA reaction of oxadiazole **6** with mononuclear Cu(I)-butylide **7-Cu** was performed. The positions of the ELF basin attractors and the more relevant valence basin populations of **TS11**, **IC**, and **TS12** are presented in Figure 5.

The ELF of **TS-11** shows the presence of five monosynaptic basins, V(N1), V(N2), V(C3), V(C7), and V(C8), integrating 4.08, 1.49, 0.57, 3.24, and 0.44 e, respectively. The V(N2) monosynaptic basin is associated with the creation of a non-bonding electron density at the N2 nitrogen. Note that it is not present at oxadiazole **6**, as shown in Figure 2. The two V(C3) and V(C8) monosynaptic basins created at the C3 and C8 interacting carbon, which integrate less than 1.0 e, are associated with the C3 and C8 *pseudoradical* centers demanded for the subsequent C3-C8 single bond formation [58]. Consequently, the formation of the first C3-C8 single bond has not begun at **TS11**.

The ELF of the intermediate **IC** shows the presence of four monosynaptic basins, V(N1), V’(N1), V(N2), and V(C7), integrating 2.58, 1.66, 1.96, and 3.28 e, respectively, and a new V(C3,C8) disynaptic basin integrating 1.79 e. The presence of this disynaptic basin indicates that the new C3-C8 single bond has been formed at **IC**. On the other hand, the V(N1) disynaptic basin present in **TS-11** has been separated into two monosynaptic basins; the V’(N1) disynaptic basin will be required for the further formation of the second N1-C7 single bond.

Finally, the ELF of **TS-12** shows the presence of four monosynaptic basins, V(N1), V’(N1), V (N2), and V(C7), integrating 2.67, 1.43, 2.05, and 3.13 e, respectively, and the V(C3,C8) disynaptic basin integrating 1.85 e. The population of the V(N2) monosynaptic basin has been increased to 2.05 e. Therefore, there is a high similarity between the electronic structure of **IC** and **TS-12**. Only some minor changes in basin populations are observed. These behaviors characterize the earlier character of **TS-12**.

### 2.3. Molecular Docking Analysis

Prior research has demonstrated that sulfonamides and sydnones exhibit anti-inflammatory and antiviral properties [64]. Moreover, recent data have revealed the possibility to target viral proteins with a sulfonamide–sydnone hybrid [65]. These considerations prompted us to investigate the interactions between the six ligands **5a**–**f** and the two viral proteins cyclophilin A (6GJI) and gp120 (6L1Y) using molecular docking. In both cases, the best binding configurations were determined, and the corresponding binding energies were calculated (Table 3). All six compounds can bind reasonably well to cyclophilin A (CypA). The docking suggests broadly comparable binding affinities across the series, with **5d** and **5f** showing slightly more favorable scores within the limitations of the method. The extent of binding was found to be more variable with the gp120 protein, with compounds **5c** and **5e** identified as weak binders compared to **5d,** which is the best binder in the series. Representative models of the interaction between compound **5d** and the two proteins are presented in Table 4. The binding configurations are a little distinct from one compound to another. For example, the interaction **5d** with the CypA includes six van der Waals interactions and two H-bond interactions, whereas **5f** with CypA involves three van der Waals interactions and only one hydrogen interaction. Nevertheless, all six compounds were able to form stable complexes with CypA. In the case of the gp120 protein, the best configuration was observed with compound **5d** which engaged two van der Waals interactions and one hydrogen interaction with the protein. A summary of the binding interactions is given in Table 5.

### 2.4. Physicochemical Properties

**Figure 6:**
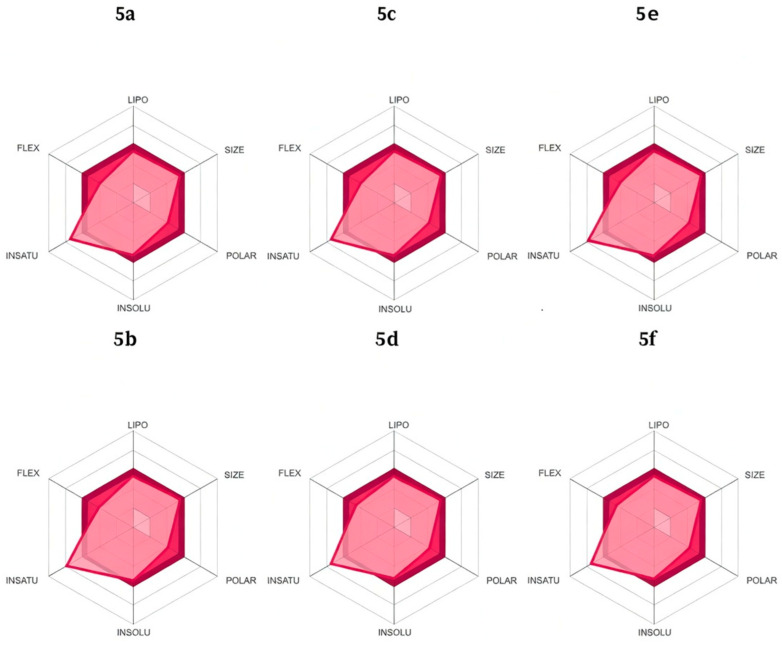
Metabolic properties of **5a–f.**

The physicochemical properties of the compounds have been analyzed (Figure 6)**.** All six compounds **5a–f** exhibit a molecular weight (MW) of less than 500 Da, fewer than 10 rotatable bonds, fewer than 10 hydrogen bond acceptors, and fewer than five hydrogen bond donors. Additionally, the topological polar surface area (TPSA) for each compound is below 140 Å^2^. Larger molecular weights (>500 Da), excessive hydrogen bonding, or high lipophilicity can impede a compound’s ability to penetrate biological membranes, thereby reducing its bioavailability. Similarly, a higher number of rotatable bonds and a larger TPSA value can negatively affect the bioavailability by compromising membrane permeability [66]. But in the present case, no atypical molecular characteristic was observed.

Regarding lipophilicity, the logP value, specifically XLOGP3 (which ranges from 1 to 4), is indicative of good absorption for oral drug candidates [67]. Lower logP values generally improve the solubility in aqueous environments, enhancing dissolution, while higher values improve membrane permeability, facilitating better absorption across lipid membranes. Among the six ligands assessed, only **5e** and **5f** have XLOGP3 values below 4, with values of 3.78 and 3.88, respectively (Figure 6). These two compounds present satisfactory physico-chemical properties.

The ability of a compound to penetrate the blood–brain barrier (BBB) is typically correlated with its LogP value. Compounds with moderate LogP values are generally associated with better BBB permeability [67]. However, none of the six ligands appear to effectively cross the BBB, as their LogP values are relatively low, making it challenging for them to penetrate this barrier, as shown in Figure 6.

In terms of solubility, the Log S value was predicted using the ESOL model. A Log S value of less than −4 indicates poor solubility, which can significantly affect the bioavailability due to inadequate amounts of the drug being available in the systemic circulation [67]. In this case, all six ligands display low solubility, as their Log S values are all below −4, as shown in Table 6.

### 2.5. Metabolism and Toxicity Prediction

From a metabolism standpoint, **5a** was predicted to inhibit several cytochrome P450 (CYP) enzymes, including CYP2C19, CYP2C9, CYP2D6, and CYP3A4. In contrast, **5b**, **5c**, and **5d** were predicted to inhibit CYP2C19, CYP2C9, and CYP3A4. Furthermore, **5e** and **5f** were identified as potential P-glycoprotein (Pgp) substrates, in addition to inhibiting CYP2C19, CYP2C9, CYP2D6, and CYP3A4. The inhibition of these key CYP enzymes, particularly CYP2C19, CYP2C9, CYP2D6, and CYP3A4, raises concerns about potential drug–drug interactions (DDIS). These interactions could significantly alter the metabolism of not only the ligands themselves but also other concurrently administered medications, leading to altered drug levels, which may result in reduced therapeutic efficacy or increased toxicity. Moreover, the inhibition of multiple CYP enzymes and Pgp could further complicate the pharmacokinetics of other drugs that rely on these pathways for metabolism and clearance, as shown in Table 7. The computational analysis suggests that the compounds could negatively impact the functioning of diverse CYP enzymes. This potential needs to be taken into account for subsequent in vivo evaluation.

The toxicity of the ligands was also evaluated. It was predicted that **5a**, **5b**, **5e**, and **5f** would exhibit lower toxicity compared to **5c** and **5d**. Specifically, the predicted LD_50_ values for **5a**, **5b**, **5e**, and **5f** are 1000 mg/kg, while those for **5c** and **5d** are predicted to be significantly lower at 300 mg/kg, indicating a higher potential for toxicity, as shown in Table 7. 

The in silico analyses suggest that the studied compounds present favorable target-binding capacities and satisfactory ADMET properties (acceptable Lipinski parameters), which augur well for future experimental measurements. These data are encouraging, yet the limitations cannot be under-estimated. The low predicted solubility and possible CYP-related liabilities need to be considered for future drug design in the series. Molecules of interest have been designed, but it remains an entire challenge to extend the hit-to-lead optimization process and to qualify in vivo active molecules with a robust pharmaceutical profile. The drug design work must continue. 

## 3. Materials and Methods

### 3.1. General Remarks

Melting points were measured using a Köfler bench apparatus (Wagner & Munz GmbH, Munich, Germany). The reactions were monitored by thin layer chromatography (TLC) using aluminum silica gel plates (silica gel 60, F 254 Merck 0.063–0.200 mm) (Merck KGaA, Darmstadt, Germany). Spots were located using UV light (254 nm, 365 nm). ^1^H NMR and ^13^C NMR spectra were recorded in DMSO-*d_6_* with TMS as an internal reference using a Bruker AC 500 (^1^H) or 126 MHz (^13^C) device. NMR multiplicities are abbreviated as follows: s = singlet, d = doublet, q = quartet, m = multiple. Chemical shifts are given in δ parts per million (ppm), and coupling constants (J) are expressed in Hz. High-resolution mass spectra (HRMS) were recorded on a Waters Q-TOF instrument (PAHRB, University of La Rochelle). Analyses were carried out using a high-resolution mass spectrometer (XEVO G2-S QTof) equipped with an ElectroSpray Ionization (ESI) source (Waters, Manchester, UK). Compounds were detected by infusion of samples at a concentration of 1 µg/mL as ions in both positive and negative polarities. Samples are prepared as follows: 10 mg of each compound in 1 mL of DMSO (C = 10 mg/mL); shaking and centrifugation for 5 min at 13,000 rpm; dilution to 1 µg/mL in H2O/ACN (50:50; *v*/*v*). The MS parameters were acquisition polarities: negative and positive; infusions at 5 µL/min; capillary voltage = 3 kV (+) and 2.5 kV (−), temperatures = 120°C for source and 500°C for desolvation gas, gas flow: 50 L/h for cone gas and 800 L/h for desolvation gas, mass correction with leucine enkephalin at 1 ng/µL.

### 3.2. Synthesis 

General procedure for *N*-alkyation of *N*-(2,3-dihydro-1,4-benzodioxin-6-yl)-benzenesulfonamide derivatives **(4a**–**c)** with propargyl bromide (**GP1**).

To a solution of *N*-(2,3-dihydro-1,4-benzodioxin-6-yl)-benzenesulfonamides **3a**–**c** (100 mg, 0.32 mmol) in acetone (10 mL), potassium carbonate (3 eq., 133 mg, 0.96 mmol) was added. After 5 min, propargyl bromide (1.2 eq., 0.03 mL, 0.38 mmol) was added drop wise, and the reaction was stirred at room temperature until the complete consumption of the starting material (4 h). The resulting products were obtained as white precipitates by the slow addition of water. The precipitates were filtered out and air-dried to yield he desired compounds.

*N*-(2,3-Dihydrobenzo[b][1,4]dioxin-6-yl)-4-methyl-*N*-(prop-2-yn-1-yl)benzenesulfonamide (**4a**). Yield: 97% (106 mg); white solid; R_f_: 0.42; m.p.:100–102 °C. ^1^H NMR (500 MHz, DMSO-*d_6_*) δ 7.53 (d, *J* = 8.3 Hz, 2H), 7.38 (d, *J* = 8.0 Hz,2H), 6.81 (d, *J* = 8.7 Hz,1H), 6.64 (d, *J* = 2.5 Hz,1H), 6.57 (dd, *J* = 8.7, 2.5 Hz,1H), 4.41 (d, *J* = 2.4 Hz, 2H), 4.23 (q, J = 4.9 Hz,4H), 3.23 (s,1H), 2.39 (s,3H). ^13^C NMR (126 MHz, DMSO-*d_6_*) δ 143.72, 143.34, 143.02, 135.25, 129.68(2C), 127.52(2C), 121.31, 117.26, 117.05, 78.68, 76.26, 64.06, 63.99, 40.97, 21.10. MALDI-HRMS: m/z calcd. for C_18_H_17_NO_4_S ([M + H]^+^): 343.0878; found: 344.0947.

4-Chloro-*N*-(2,3-dihydrobenzo[b][1,4]dioxin-6-yl)-*N*-(prop-2-yn-1-yl)benzenesulfonamide (**4b**). Yield: 95% (107mg); white solid; Rf:0.44; m.p.:102–104 °C. ^1^H NMR (500 MHz, DMSO-*d_6_*) δ 7.72–7.58 (m, 4H), 6.83 (d, *J* = 8.6 Hz,1H), 6.67 (d, *J* = 2.5 Hz, 1H), 6.60 (dd, *J* = 8.7, 2.5 Hz, 1H), 4.44 (d, *J* = 2.4 Hz, 2H), 4.24 (q, *J* = 4.8 Hz, 4H), 3.26 (s, 1H). ^13^C NMR (126 MHz, DMSO-*d_6_*) δ 143.51, 143.08, 138.25, 136.85, 131.35, 129.44 (2C), 129.37 (2C), 121.35, 117.29, 117.15, 78.41, 76.49, 64.05, 63.97, 41.11.

*N*-(2,3-Dihydrobenzo[b][1,4]dioxin-6-yl)-4-methoxy-*N*-(prop-2-yn-1-yl)benzenesulfonamide (**4c)**. Yield: 93% (100mg); white solid; R_f_:0.37; m.p.:84–86 °C. ^1^H NMR (500 MHz, DMSO-*d_6_*) δ 7.65–7.46 (m, 2H), 7.17–7.01 (m, 2H), 6.81 (d, *J* = 8.6 Hz, 1H), 6.65 (d, *J* = 2.5 Hz, 1H), 6.58 (dd, *J* = 8.6, 2.5 Hz, 1H), 4.38 (d, *J* = 2.4 Hz, 2H), 4.23 (q, *J* = 4.8 Hz, 4H), 3.84 (s, 3H), 3.22 (s, 1H) ppm. ^13^C NMR (126 MHz, DMSO-*d_6_*) δ 162.77, 143.28, 142.99, 131.80, 129.73(2C), 129.58(2C), 121.24, 117.23, 116.99, 114.31, 78.73, 76.19, 64.04, 63.96, 55.72, 40.75.

General Procedure for 1,3-dipolar cycloaddition of pyrazoles with terminal alkynes (**GP2**).

Sydnone (50 mg, 0.3 mmol), *N*-(2,3-dihydrobenzo[b][1,4]dioxin-6-yl)-4-methyl-*N*-(prop-2-yn-1-yl)benzenesulfonamide (112 mg, 0.3 mmol), triethylamine (1 equiv., 43 μL, 0.3 mmol), copper(II) sulphate pentahydrate (0.2 equiv., 15 mg, 0.06 mmol), sodium ascorbate (1 equiv., 57 mg, 0.3 mmol) and 1,10-phenanthroline (0.2 equiv., 11 mg, 0.06 mmol) were dissolved in a mixture of water (1.0 mL) and *t*-BuOH (1.0 mL), respectively. After complete conversion of the starting materials, the reaction was diluted and extracted with CH_2_Cl_2_ (3 × 10 mL). The organic layer was then dried over Na_2_SO_4_, filtered out, and concentrated under reduced pressure. The crude products were purified by column chromatography on silica gel (Hexane: EtOAc 70:30).

*N*-(2,3-Dihydrobenzo[b][1,4]dioxin-6-yl)-4-methyl-*N*-((1-phenyl-1*H*-pyrazol-4-yl)methyl) benzenesulfonamide (**5a**). Yield: 90% (125mg); white solid; R_f_: 0.47; m.p.:172–174 °C. ^1^H NMR (500 MHz, DMSO-*d_6_*) δ 8.29 (s, 1H), 7.73 (d, *J* = 7.7 Hz, 2H), 7.56 (d, *J* = 8.3 Hz, 2H), 7.50–7.38 (m, 5H), 7.27 (t, *J* = 7.4 Hz, 1H), 6.73 (d, *J* = 8.6 Hz, 1H), 6.59 (d, *J* = 2.5 Hz, 1H), 6.47 (dd, *J* = 8.7, 2.5 Hz, 1H), 4.64 (s, 2H), 4.18 (q, *J* = 4.7 Hz, 4H), 2.41 (s, 3H) ppm. ^13^C NMR (126 MHz, DMSO-*d_6_*) δ 143.52, 142.96, 142.91, 140.83, 139.37, 134.89, 131.48, 129.72(2C), 129.52(2C), 127.49(2C), 126.90, 126.17, 121.35, 118.53, 118.01(2C), 117.71, 116.78, 63.94, 63.87, 44.89, 20.99. MALDI-HRMS: *m*/*z* calcd. for C_25_H_23_N_3_O_4_S ([M + H]^+^): 461.1410; found: 462.1488.

4-Chloro-*N*-(2,3-dihydrobenzo[b][1,4]dioxin-6-yl)-*N*-((1-phenyl-1H-pyrazol-4-yl)methyl) benzenesulfonamide (**5b**). Yield: 92 % (133mg); white solid; R_f_: 0.46; m.p.:174–176 °C. ^1^H NMR (500 MHz, DMSO-*d_6_*) δ 8.31 (s, 1H), 7.76–7.65 (m, 6H), 7.49 (s, 1H), 7.48–7.43 (m, 2H), 7.31–7.25 (m, 1H), 6.75 (d, *J* = 8.6 Hz, 1H), 6.62 (d, *J* = 2.5 Hz, 1H), 6.50 (dd, *J* = 8.7, 2.5 Hz, 1H), 4.67 (s, 2H), 4.19 (d, *J* = 2.0 Hz, 4H) ppm. ^13^C NMR (126 MHz, DMSO-*d_6_*) 143.13, 143.00, 140.84, 139.35, 138.10, 136.51, 131.10, 129.53(2C), 129.45(2C), 129.37(2C), 126.97, 126.21, 121.42, 118.32, 118.04(2C), 117.79, 116.91, 63.95, 63.88, 45.03. MALDI-HRMS: *m*/*z* calcd. for C_24_H_20_N_3_O_4_SCl ([M + H]^+^): 481.0864; found: 482.0959.

*N*-(2,3-Dihydrobenzo[b][1,4]dioxin-6-yl)-4-methoxy-*N*-((1-phenyl-1*H*-pyrazol-4-yl)methyl) benzenesulfonamide (**5c**). Yield: 90 % (130mg); weight solid; R_f_: 0.36; m.p.:158–160 °C. ^1^H NMR (500 MHz, DMSO-*d_6_*) δ 8.29 (s, 1H), 7.73 (dd, *J* = 8.7, 1.1 Hz, 2H), 7.63–7.57 (m, 2H), 7.48 (s, 1H), 7.47–7.43 (m, 2H), 7.30–7.24 (m, 1H), 7.16–7.11 (m, 2H), 6.73 (d, *J* = 8.7 Hz, 1H), 6.60 (d, *J* = 2.5 Hz, 1H), 6.47 (dd, *J* = 8.7, 2.5 Hz, 1H), 4.63 (s, 2H), 4.18 (s, 4H), 3.86 (s, 3H) ppm. ^13^C NMR (126 MHz, DMSO-*d_6_*) δ 162.68, 142.92, 142.90, 140.84, 139.38, 131.62, 129.68(2C), 129.52(2C), 129.29(2C), 126.89, 126.16, 121.32, 118.56, 118.00(2C), 117.76, 116.75, 114.40, 63.94, 63.87, 55.72, 44.80. MALDI-HRMS: *m*/*z* calcd. for C_25_H_23_N_3_O_5_S ([M + H]^+^): 477.1359; found: 478.1444.

The compounds **5d–f** were prepared according to the general procedure (**GP2**) using 3-(4-fluorophenyl)-1,2,3-oxadiazol-3-ium-5-olate (1 equiv., 50 mg, 0.28 mmol) and were isolated by column chromatography on silica gel (Hexane: EtOAc 70:30).

*N*-(2,3-Dihydrobenzo[b][1,4]dioxin-6-yl)-*N*-((1-(4-fluorophenyl)-1*H*-pyrazol-4-yl)methyl)-4-methylbenzenesulfonamide (**5d**). Yield: 96% (129 mg); white solid; R_f_: 0.35; m.p.:132–134°C. ^1^H NMR (500 MHz, DMSO-*d_6_*) δ 8.28 (s, 1H), 7.81–7.72 (m, 2H), 7.58–7.50 (m, 2H), 7.47 (s, 1H), 7.42 (d, *J* = 8.3 Hz, 2H), 7.34–7.26 (m, 2H), 6.73 (d, *J* = 8.7 Hz, 1H), 6.59 (d, *J* = 2.5 Hz, 1H), 6.47 (dd, *J* = 8.6, 2.5 Hz), 1H, 4.63 (s, 2H), 4.18 (d, *J* = 2.2 Hz, 4H), 2.41 (s, 3H) ppm. ^13^C NMR (126 MHz, DMSO-*d_6_*) δ 159.20, 143.52, 142.97, 142.91, 140.87, 135.99, 134.88, 131.48, 129.72(2C), 127.49(2C), 127.11, 121.35, 120.07, 120.00, 118.63, 117.71, 116.78, 116.32, 116.13, 63.94, 63.87, 44.89, 20.99. MALDI-HRMS: *m*/*z* calcd. for C_25_H_22_N_3_O_4_SF ([M + H]^+^): 479.1316; found: 480.1401.

4-Chloro-*N*-(2,3-dihydrobenzo[b][1,4]dioxin-6-yl)-*N*-((1-(4-fluorophenyl)-1*H*-pyrazol-4-yl)methyl)benzenesulfonamide (**5e**). Yield: 93% (130 mg); white solid; R_f_: 0.4; m.p.:142-144°C. ^1^H NMR (500 MHz, DMSO-*d_6_*) δ 8.31 (s, 1H), 7.82–7.73 (m, 2H), 7.73–7.64 (m, 4H), 7.49 (s, 1H), 7.35–7.27 (m, 2H), 6.75 (d, *J* = 8.7 Hz, 1H), 6.63 (d, *J* = 2.5 Hz, 1H), 6.50 (dd, *J* = 8.7, 2.5 Hz, 1H), 4.67 (s2H), 4.19 (s, 4H) ppm. ^13^C NMR (126 MHz, DMSO-*d_6_*) δ 159.21, 143.14, 143.00, 140.87, 138.10, 136.51, 135.97, 131.10, 129.45(2C), 129.37(2C), 127.17, 121.42, 120.10, 120.03, 118.42, 117.79, 116.91, 116.32, 116.14, 63.96, 63.88, 45.03. MALDI-HRMS: m/z calcd. for C_24_H_19_N_3_O_4_SFCl ([M + Na]^+^): 499.0770; found: 522.0679.

*N*-(2,3-dihydrobenzo[b][1,4]dioxin-6-yl)-*N*-((1-(4-fluorophenyl)-1*H*-pyrazol-4-yl)methyl)-4-methoxybenzenesulfonamide (**5f**). Yield: 90% (121 mg); white solid; R_f_: 0.38; m.p.:144-146°C. ^1^H NMR (500 MHz, DMSO-*d_6_*) δ 8.24 (s, 1H), 7.78–7.65 (m, 2H), 7.62–7.49 (m, 2H), 7.43 (s1H), 7.32–7.18 (m, 2H), 7.16–6.99 (m, 2H), 6.69 (d, *J* = 8.6 Hz, 1H), 6.56 (d, *J* = 2.5 Hz, 1H), 6.43 (dd, *J* = 8.6, 2.5 Hz, 1H), 4.58 (s, 2H), 4.15 (s, 4H), 3.82 (s, 3H) ppm. ^13^C NMR (126 MHz, DMSO-*d_6_*) δ 162.74, 159.23, 142.93, 142.91, 140.87, 136.01, 131.56, 129.67(2C), 129.28(2C), 127.09, 121.32, 120.06, 119.99, 118.67, 117.77, 116.76, 116.32, 116.14, 114.40, 63.95, 63.87, 55.72, 44.80. MALDI-HRMS: *m*/*z* calcd. for C_25_H_22_N_3_O_5_SF ([M + Na]^+^): 495.1265; found: 518.1179.

### 3.3. In Silico Methods

#### 3.3.1. Computational Details

This MEDT study was conducted using chemical calculations within the framework of Density Functional Theory (DFT) [68]. The *ω*B97X-D [69] exchange-correlation functional and with the standard 6-311G(d,p) basis set [70], which incorporates d-type polarization on second-row elements as well as p-type polarization functions on hydrogen atoms, were used throughout this MEDT study. TSs were verified by the presence of a single imaginary frequency. Geometry optimizations were performed using the Berny algorithm [71,72]. The intrinsic reaction coordinate (IRC) approach [73] was applied to confirm the unique connectivity between each TS and its associated minima [74,75]. Solvent effects were incorporated by fully reoptimizing the gas-phase stationary points at the same theoretical level using the polarizable continuum model (PCM) [76,77] within the self-consistent reaction field (SCRF) framework [78,79,80], with 2-butanol, ε = 17.8, as the solvent.

GEDT values [60] were evaluated according to the expression GEDT(f) = Σq_f_, where q corresponds to the natural atomic charges [81,82] of the atoms defining each framework (f) at the TS geometries. 

The global electrophilicity ω index [60], is given by the following expression:

ω = (μ^2^/2η) (1)

in terms of the electronic chemical potential μ and the chemical hardness η. Both quantities may be approached in terms of the one-electron energies of the frontier molecular orbital HOMO and LUMO, ε_H_ and ε_L_, as μ ≈ (ε_H_ + ε_L_)/2, and η ≈ (ε_L_ + ε_H_), respectively [57].

The empirical (relative) nucleophilicity *Ν* index [61], based on the HOMO energies obtained within the Kohn-Sham scheme, is defined as

*Ν* = ε_H_ (Nu) − ε_H_ (TCE) (2)

where tetracyanoethylene (TCE) is the reference. 

All quantum chemical calculations were carried out with the Gaussian 16 software package [83]. ELF analyses [53] of the ωB97X-D/6-311G(d,p) single-determinant wavefunctions were performed using the TopMod program [84] on a cubic grid with a step size of 0.1 Bohr. Molecular structures and ELF basin attractors were visualized with GaussView 6.1 [85].

#### 3.3.2. Molecular Docking 

OpenBabel 3.0.1 software [86] was used to convert all ligands into 3D structures, followed by the steepest descent energy minimization using the MMF94 force field. The ligands were then assigned hydrogen bonds, non-polar merging, charge merging, and converted into pdbqt file format using the prepare_receptor4.py script from MGLTools version 1.5.7 [66]. The crystal structures of Cyclophilin A protein (6GJI) and gp120-CD4 protein (6L1Y) were retrieved from the Protein Data Bank (PDB). All heteroatoms were removed, hydrogen bonds were assigned, non-polar merging and charge assignments using Kollman and Gasteiger were applied, and the ligands were converted into PDBQT format [87,88]. The grid for the Cyclophilin A protein was centered at x = −0.711, y = 17.718, z = −5.897, with a grid size of x = 38, y = 36, and z = 26. For the gp120 protein, the grid was centered at x = 6.661, y = 4.103, z = −18.444, with a grid size of x = 50, y = 78, and z = 104.

#### 3.3.3. Physicochemical and Metabolic Properties 

All ligands were redrawn using ChemSketch 2024.2 software for conversion into SMILES. ADMET analysis was conducted using the Swiss ADME software SwissADME “v1.2.3” (www.swissadme.ch) from the Swiss Institute of Bioinformatics (http://www.sib.swiss, accessed on 13 March 2025) and ProTox 3.0 to predict the toxicity of ligands. The input list consists of one molecule per line, represented by simplified molecular input line entry system (SMILES) strings, and the results are displayed in tables for each molecule [89]. The bioavailability radar provides an initial assessment of the drug-like properties of the molecules of interest by considering six physicochemical characteristics: lipophilicity (LIPO), size, polarity (POLAR), insolubility (INSOLU), insaturation (INSATU), and flexibility (FLEX). Specifically, lipophilicity is measured by XLOGP3 ranging from −0.7 to +5.0, size is represented by molecular weight (MW) between 150 and 500 g/mol, polarity is indicated by the total polar surface area (TPSA) ranging from 20 to 130 Å^2^, solubility is defined by a log S value no greater than 6, saturation is based on the fraction of sp3-hybridized carbon atoms being at least 0.25, and flexibility refers to having no more than 9 rotatable bonds.

## 4. Conclusions

In summary, we have successfully synthesized 1,4-disubstituted pyrazole scaffolds through a Cu(I)-catalyzed 32CA reaction (CuSAC) involving 3-arylsydnones and *N*-(alkyl)-*N*-(2,3-dihydro-1,4-benzodioxin-6-yl)-4-benzenesulfonamides. The procedure enabled the preparation of six new compounds, structurally characterized using ^1^H and ^13^C NMR spectroscopy and mass spectrometry. It is a versatile approach for convenient access in three steps to new 1,4-disubstituted pyrazoles. The procedure reported here offers a novel approach to sulfonamides incorporating a benzodioxin unit. The mechanism of the Cu(I)-catalyzed reaction has been studied within the MEDT. This reaction is a domino process that consists in a Cu(I)-catalyzed formal 32CA reaction followed by an extrusion of CO_2_ yielding the final pyrazole. The coordination of Cu(I) cation to the anion butylide creates the mononuclear species **7-Cu**, which behaves as supernucleophile. This species reacts efficiently with the strong electrophile oxadiazole **6** in a polar reaction, reducing the activation energy of the non-catalyzed process by approximately 14 kcal·mol^−1^. The subsequent extrusion of CO_2_ is spontaneous with no appreciable barrier yielding the final pyrazole.

The pharmacology of these compounds remains to be investigated. However, preliminary evidence indicates that they may bind to viral proteins. The molecular docking analysis suggests that all six ligands can bind to both the cyclophilin A and gp120 proteins, with **5d** and **5f** showing the lowest binding energy against cyclophilin A, while **5d** exhibits a lower binding energy when bound to gp120. Compounds **5d**–**5f** deserve further investigation as anti-HIV/anti-HCV agents, as well as anti-SARS-CoV-2 [65]. The information will help the design of additional antiviral compounds in the series. Their antiviral effects will be investigated in due course.

## Figures and Tables

**Table 1:** Catalyst screening and solvent optimization for the 32CA reactions of sydnone and terminal alkynes.

Entry	Solvent	Catalyst	Additive	Time (h)	T (°C)	Yield (%)
1	Xylene	-	-	72	Reflux	-
2	Toluene	-	-	72	Reflux	-
3	DMF	CuI	-	48	Reflux	Traces
4	DMF/H_2_O (1:1)	CuSO_4_.5H_2_O	Na asc.	48	80	30%
5	EtOH/H_2_O (1:1)	CuSO_4_.5H_2_O	Na asc.	48	80	-
6	DCM/H_2_O (1:1)	CuSO_4_.5H_2_O	Na asc.	48	60	-
7	Isopropanol/H_2_O (1:1)	CuSO_4_.5H_2_O	Na asc.	72	60	-
**8**	***t*-BuOH/H_2_O** (1:1)	**CuSO_4_.5H_2_O**	**Na asc.**	**1**–**4**	**60**–**80**	**96%**
9	*t*-BuOH/H_2_O (1:1)	CuSO_4_.5H_2_O	Na asc.	24	25	12%
10	H_2_O	CuSO_4_.5H_2_O	Na asc.	72	60	-

Na asc. = Sodium ascorbate.

**Table 2:** B3LYP/6-31G(d) electronic chemical potential *μ*, chemical hardness *η*, electrophilicity ω, and nucleophilicity *N* indices, in eV, of oxadiazole **6**, butyne **7**, and mononuclear Cu(I)-butylide **7-Cu**.

	μ	η	ω	*N*
Oxadiazole **6**	−4.13	3.84	2.22	3.07
Cu(I)-butylide **7-Cu**	−3.03	4.09	1.12	4.05
Butyne **7**	−2.73	8.57	0.44	2.10

**Table 3:** Molecular docking score for compounds **5a–f** bound to CypA and gp120.

Ligands	Cyclophilin A (kcal/mol)	gp120-CD4 (kcal/mol)
**5a**	−8.4	−9.1
**5b**	−8.8	−9.0
**5c**	−8.4	−6.8
**5d**	−8.9	−9.7
**5e**	−8.1	−7.7
**5f**	−8.9	−9.0

**Table 4:** Example of binding map for protein–ligand complex.

Ligand	Cyclophilin A Protein
**5d**	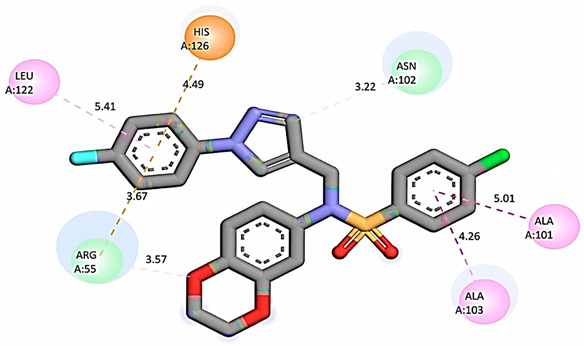
	**gp120-CD4 Protein**
	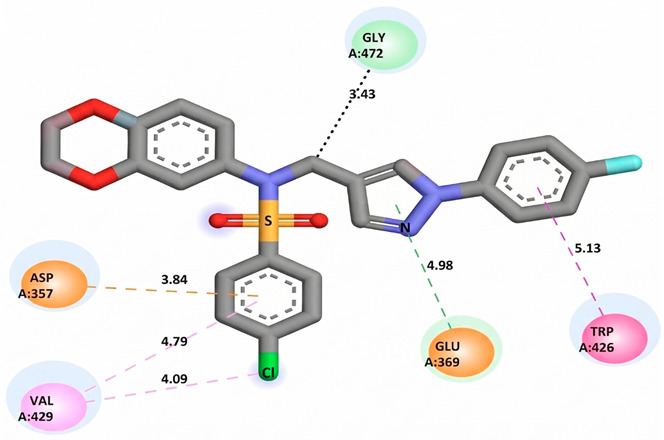

**Table 5:** Hydrogen bond and hydrophobic interactions implicated in the ligand–protein complexes.

Proteins	Cyclophilin A		gp120-CD4	
Ligands	H-bonds	Hydrophobic Interactions	H-bonds	Hydrophobic Interactions
**5a**	-	ALA101 ASN102 ALA103 PHE113 LEU122 HIS126	GLY472	ASP367 TRP426 VAL429
**5b**	HIS126	HIS54 ARG55 ALA101 ALA103 LEU122	MET425 GLY472	TRP426 VAL429 VAL370
**5c**	ARG55	ALA101 ALA103 LEU122 HIS126	THR283 ASP473	LYS282 VAL370 VAL454
**5d**	ARG55 ASN102	LEU122 ALA101 ALA103	GLY472	TRP426 VAL429
**5e**	HIS54	LEU122 ALA101 ASN102 ALA103 PHE113 HIS126	VAL281 LYS282 THR283 SER363 GLY365 HLY471	TRP96 VAL370 VAL454
**5f**	ARG55	ALA101 ALA103 PHE113 LEU122	ASP367 ASN424 ARG475	VAL370 MET425 TRP426

**Table 6:** Physico-chemical properties of **5a**–**f**.

	Physico-Chemical Properties					Lipophilicity	Drug- likeness	BBB Permeability	Bioavailability Score
Compounds	MW (g/mol)	Rotatable Bonds	H-bond Acceptor	H-bond Donor	TPSA	LogP(o/w) (XLOGP3)	Lipinski Violation		
**5a**	461.53	6	5	0	82.04	4.17	YES	No	0.55
**5b**	479.52	6	6	0	82.04	4.27	YES	No	0.55
**5c**	481.95	6	5	0	82.04	4.44	YES	No	0.55
**5d**	499.94	6	6	0	82.04	4.54	YES	No	0.55
**5e**	477.53	7	6	0	91.27	3.78	YES	No	0.55
**5f**	495.52	7	7	0	91.27	3.88	YES	No	0.55

**Table 7:** Pharmacokinetic properties for **5a**–**f**.

	Water Solubility	Pharmacokinetics							Toxicity (ProTox-III Server)	
Compounds	Log S (ESOL)	GI Absorption	Pgp Substrate	CYP1A2 Inhibitor	CYP2C1G Inhibitor	CYP2CG Inhibitor	CYP2D6 Inhibitor	CYP3A4 Inhibitor	Predicted LD50 (mg/kg)	Toxicity Class 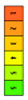
**5a**	−5.45	High	No	No	Yes	Yes	Yes	Yes	1000 mg/kg	4
**5b**	−5.61	High	No	No	Yes	Yes	No	Yes	1000 mg/kg	4
**5c**	−5.75	High	No	No	Yes	Yes	No	Yes	300 mg/kg	3
**5d**	−5.9	High	No	No	Yes	Yes	No	Yes	300 mg/kg	3
**5e**	−5.22	High	Yes	No	Yes	Yes	Yes	Yes	1000 mg/kg	4
**5f**	−5.38	High	Yes	No	Yes	Yes	Yes	Yes	1000 mg/kg	4

## Data Availability

All data are contained within the article and its Supplementary Materials.

## Supplementary Materials

The following supporting information can be downloaded at: https://www.mdpi.com/article/10.3390/molecules31081250/s1, Figure S1: ^1^H NMR spectrum of compound **4a** in DMSO-*d_6_*; Figure S2. 13C NMR spectrum of compound **4a** in DMSO-d6; Figure S3. 13C NMR DEPT 135 spectrum of compound **4a** in DMSO-d6; Figure S4. MS spectrum of compound **4a**; Figure S5. 1H NMR spectrum of compound **4b** in DMSO-d6; Figure S6. 13C NMR spectrum of compound **4b** in DMSO-d6; Figure S7. ^13^C NMR DEPT 135 spectrum of compound **4b** in DMSO-*d_6_*; Figure S8. ^1^H NMR spectrum of compound **4c** in DMSO-*d_6_*; Figure S9. ^13^C NMR spectrum of compound **4c** in DMSO-*d_6_*; Figure S10. ^13^C NMR DEPT 135 spectrum of compound **4c** in DMSO-*d_6_*; Figure S11. ^1^H NMR spectrum of compound **5a** in DMSO-*d_6_*; Figure S12. ^13^C NMR spectrum of compound **5a** in DMSO-*d_6_*; Figure S13. ^13^C NMR DEPT 135 spectrum of compound **5a** in DMSO-*d_6_*; Figure S14. MS spectrum of compound **5a**; Figure S15. ^1^H NMR spectrum of compound **5b** in DMSO-*d_6_*; Figure S16. ^13^C NMR spectrum of compound **5b** in DMSO-*d_6_*; Figure S17. ^13^C NMR DEPT 135 spectrum of compound **5b** in DMSO-*d_6_*; Figure S18. MS spectrum of compound **5b**; Figure S19. ^1^H NMR spectrum of compound **5c** in DMSO-*d_6_*; Figure S20. ^13^C NMR spectrum of compound **5c** in DMSO-*d_6_*; Figure S21. ^13^C NMR DEPT 135 spectrum of compound **5c** in DMSO-*d6*; Figure S22. MS spectrum of compound **5c**; Figure S23. ^1^H NMR spectrum of compound **5d** in DMSO-*d6*; Figure S24. ^13^C NMR spectrum of compound **5d** in DMSO-*d_6_*; Figure S25. ^13^C NMR DEPT 135 spectrum of compound **5d** in DMSO-*d_6_*; Figure S26. MS spectrum of compound **5d**; Figure S27. ^1^H NMR spectrum of compound **5e** in DMSO-*d_6_*; Figure S28. ^13^C NMR spectrum of compound **5e** in DMSO-*d_6_*; Figure S29. ^13^C NMR DEPT 135 spectrum of compound **5e** in DMSO-*d_6_*; Figure S30. MS spectrum of compound **5e**; Figure S31. ^1^H NMR spectrum of compound **5f** in DMSO-*d_6_*; Figure S32. ^13^C NMR spectrum of compound **5f** in DMSO-*d_6_*; Figure S33. ^13^C NMR DEPT 135 spectrum of compound **5f** in DMSO-*d_6_*; Figure S34. MS spectrum of compound **5f**.
