# Supplementary material for: Experimental and MEDT Study of Sydnone–Alkyne Cycloaddition-Based Synthesis of 1,4-Disubstituted Pyrazoles and In Silico Investigation of Their Binding to HCV and HIV Proteins
Source: Molecules. 2026 Apr 9;31(8):1250. doi: 10.3390/molecules31081250 (PMC13118465; doi:10.3390/molecules31081250)
Supplement: Supplementary file 1 [file molecules-31-01250-s001.zip › molecules-4216334 supplementary.pdf]

Experimental and MEDT study of sydnone-alkyne cycloaddition-based synthesis of 1,4-disubstituted pyrazoles and *in silico* investigation of their binding to HCV and HIV proteins

Souad Zerbib [1], Mohammed Eddahmi [2], Marwa Alaqarbeh [3], Pierre-Edouard Bodet [4], Valérie Thiery [5], Ahmed Fatimi [6], Natália Cruz-Martins [7], Christian Bailly [8,*], Luis R. Domingo [9,*] and Latifa Bouissane [2,*]

[1] Laboratory of Molecular Chemistry, Materials and Catalysis, Faculty of Sciences and Technologies, Sultan Moulay Slimane University, BP 523, Beni-Mellal 23000, Morocco
[2] Sustainable Processes, Advanced Materials and Computational Chemistry Team, Polydisciplinary Faculty of Beni Mellal, Sultan Moulay Slimane University, P.O. Box 592 Mghila, Beni Mellal 23000, Morocco
[3] Applied Science Research Center, Applied Science Private University, P.O. Box 11931 Jordan
[4] Plateforme d'Analyse Haute Résolution des Biomolécules PAHRB, UMR CNRS 7266 LIENSs, Université de La Rochelle, 17042 La Rochelle, France
[5] Université de La Rochelle, UMR CNRS 7266 LIENSs, Rue Olympe de Gouges, 17042 La Rochelle, France
[6] Chemical Science and Engineering Research Team, Department of Chemistry, Polydisciplinary Faculty of Beni-Mellal, Sultan Moulay Slimane University, Beni-Mellal 23000, Morocco

[7] Life and Health Sciences Research Institute (ICVS), School of Medicine, University of Minho, P.O. Box 4710-057 Braga, Portugal
[8] Institute of Pharmaceutical Chemistry Albert Lespagnol (ICPAL), Faculty of Pharmacy, University of Lille, rue du Professeur Laguesse, BP-83, F-59006 Lille, France
[9] Independent Researcher. Avd. Tirso de Molina 20, 46015 Valencia, Spain
* Correspondence: christian.bailly@univ-lille.fr (C.B.); luisrdomingo@gmail.com (L.R.D.); l.bouissane@usms.ma (L.B.)

Supplementary Materials

NMR and MS spectra of compounds 4a-c and 5a-f

Academic Editors: Naoki Kishimoto and Shiro Koseki

Received: 7 March 2026
Revised: 3 April 2026
Accepted: 4 April 2026
Published: date

Copyright: © 2026 by the authors. Submitted for possible open access publication under the terms and conditions of the Creative Commons Attribution (CC BY) license.

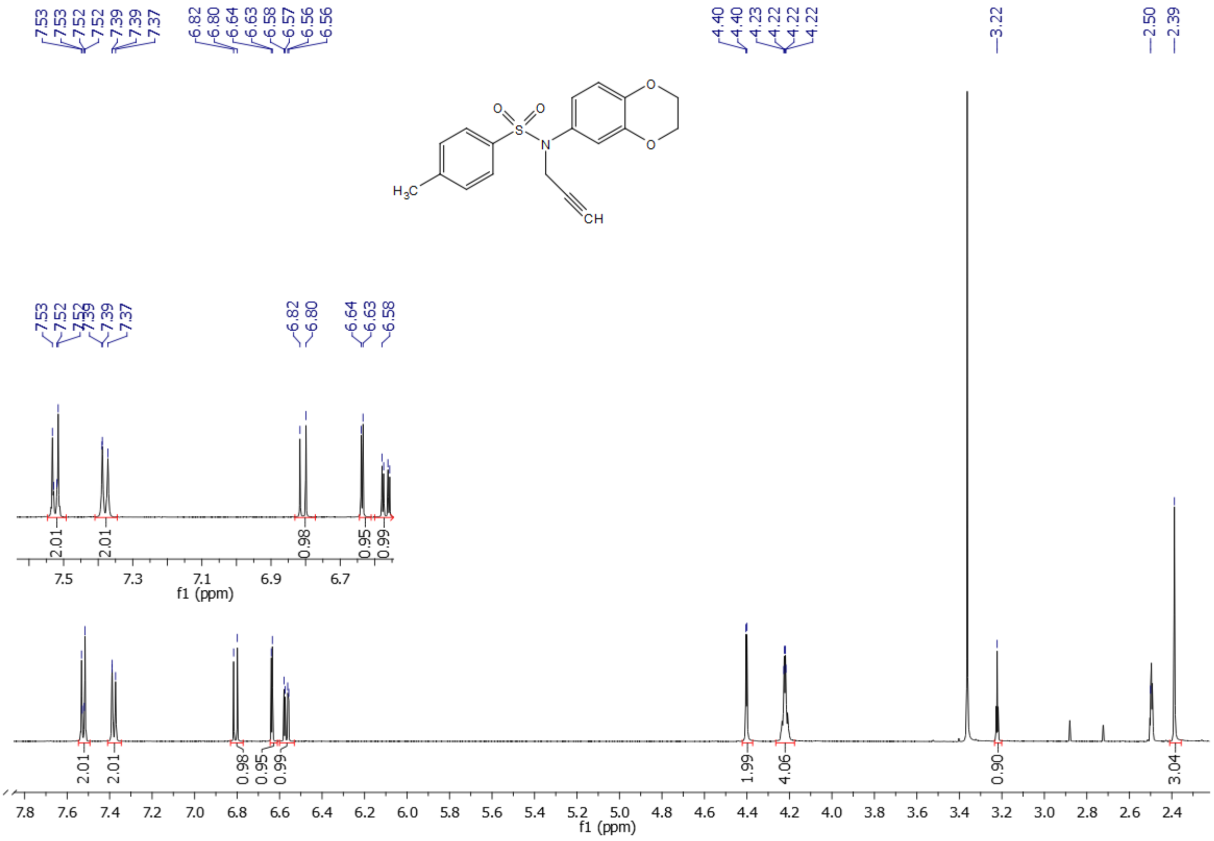

Figure S1.^{1}H NMR spectrum of compound **4a** in DMSO-d_6.

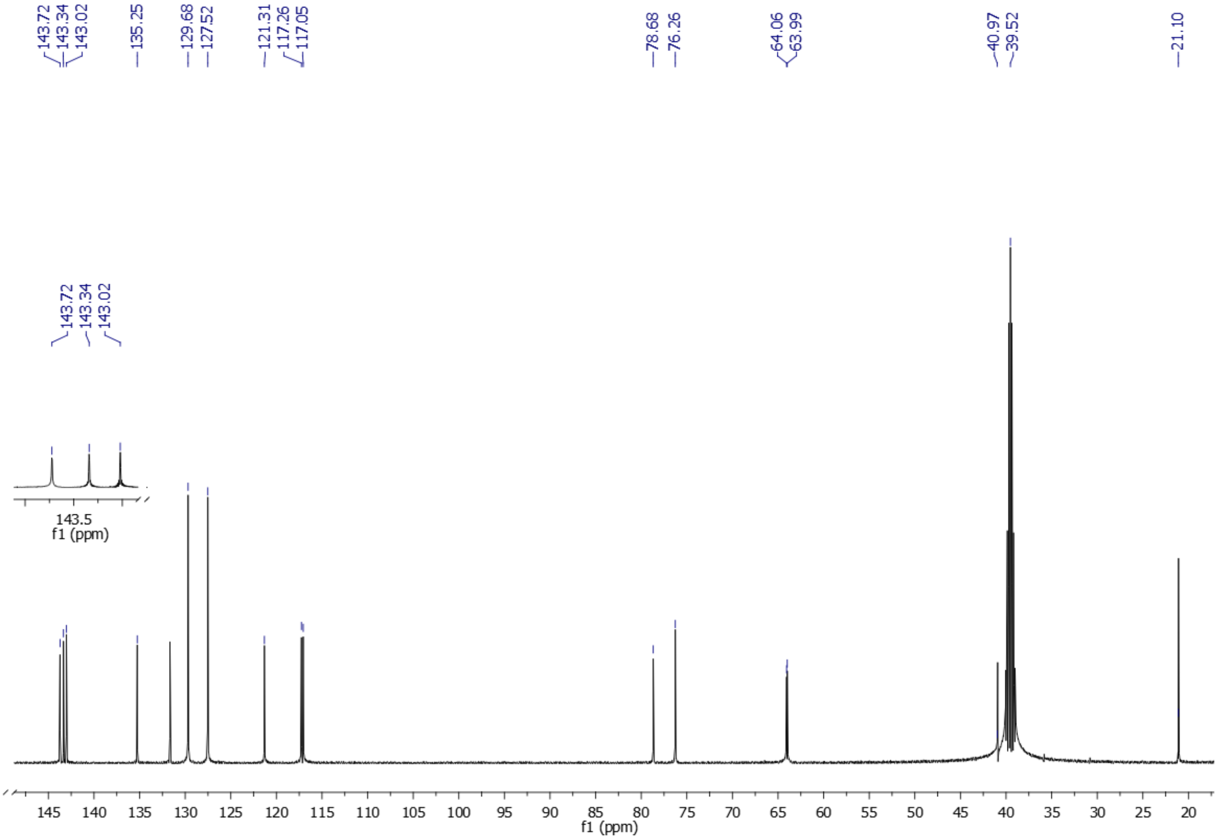

Figure S2. ^{13}C NMR spectrum of compound **4a** in DMSO-d_6.

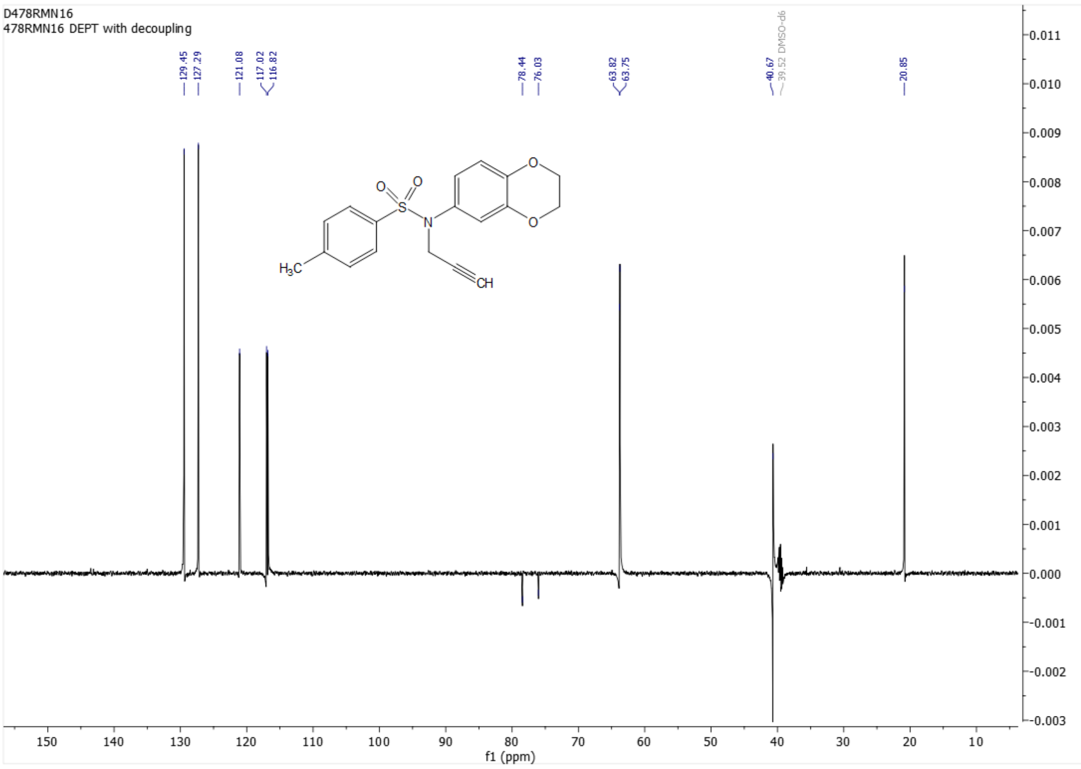

Figure S3. ^{13}C NMR DEPT 135 spectrum of compound **4a** in DMSO-d_6.

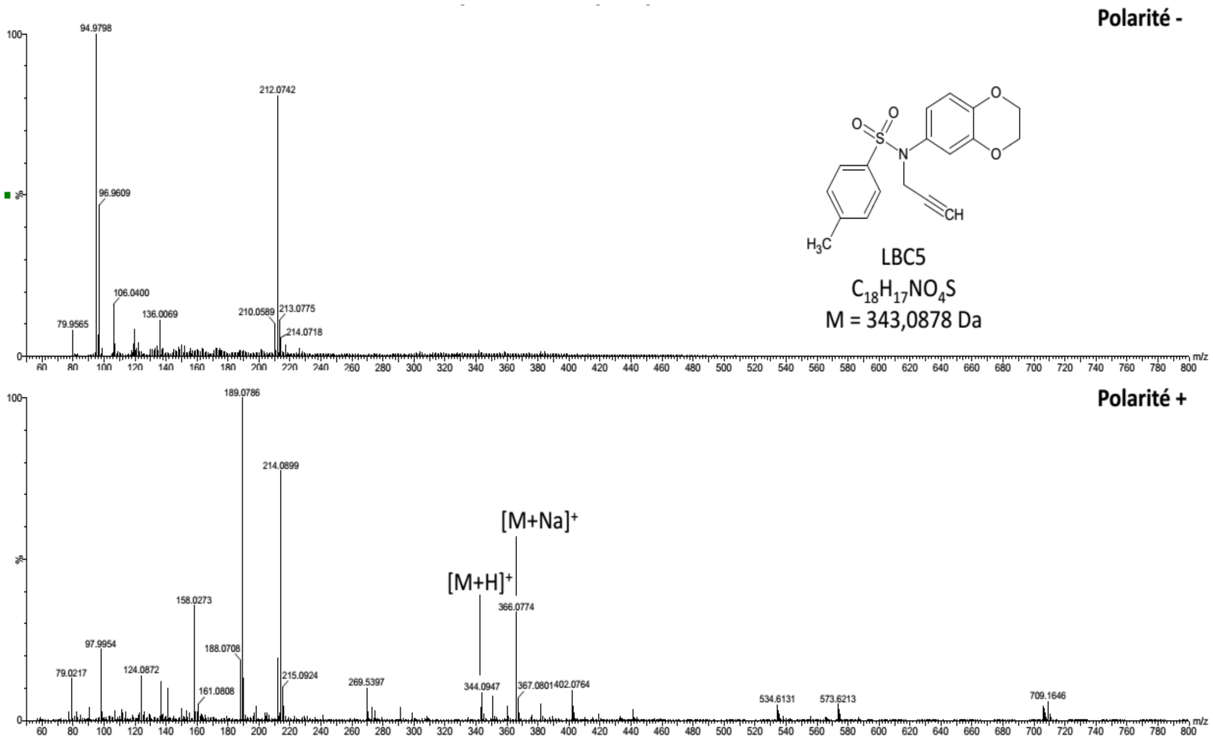

Figure S4. MS spectrum of compound **4a**

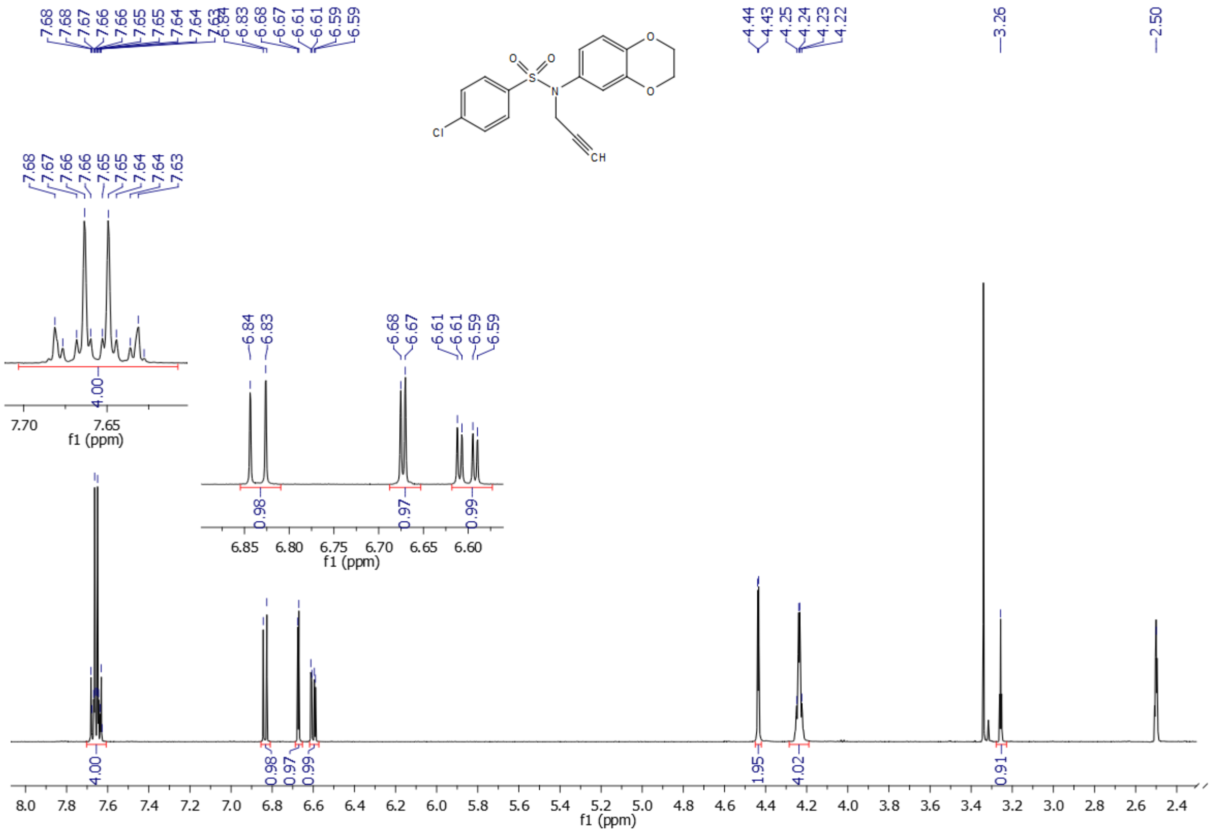

Figure S5. ^{1}H NMR spectrum of compound **4b** in DMSO-d_6.

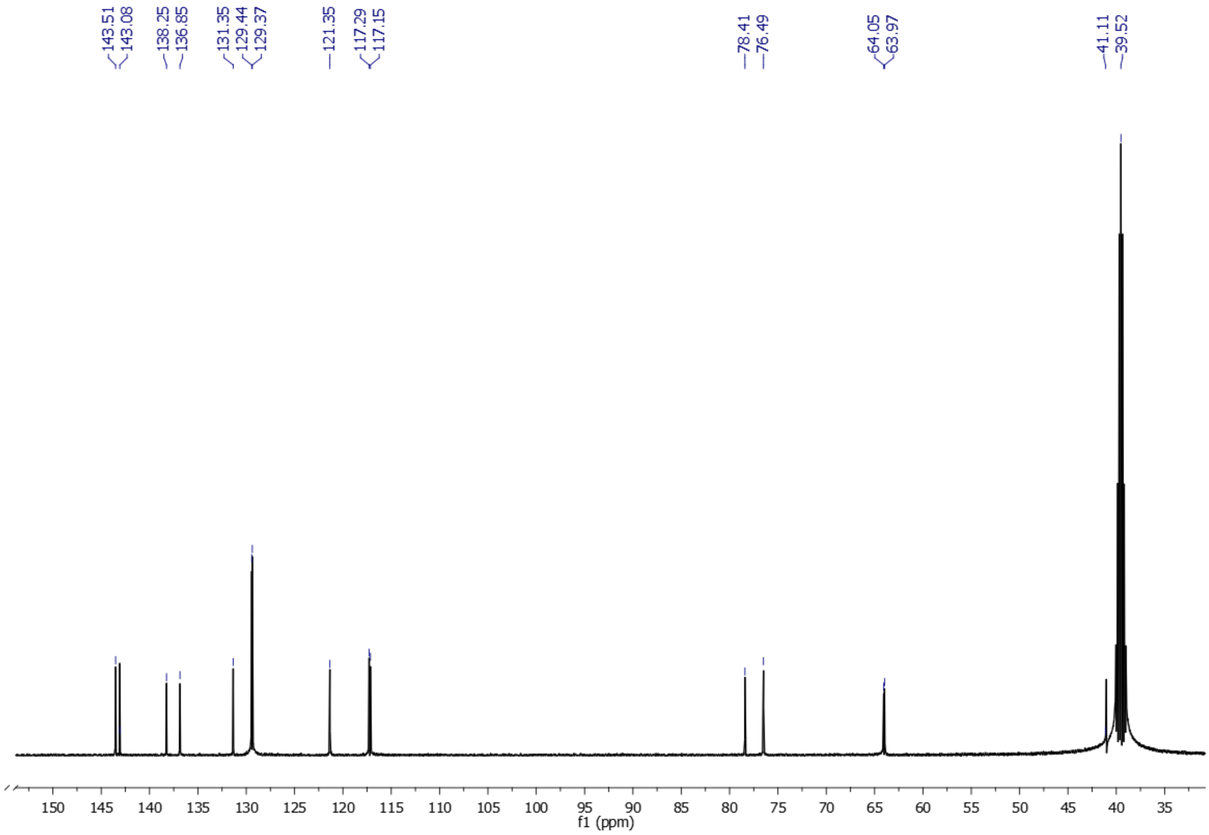

Figure S6. ^{13}C NMR spectrum of compound **4b** in DMSO-d_6.

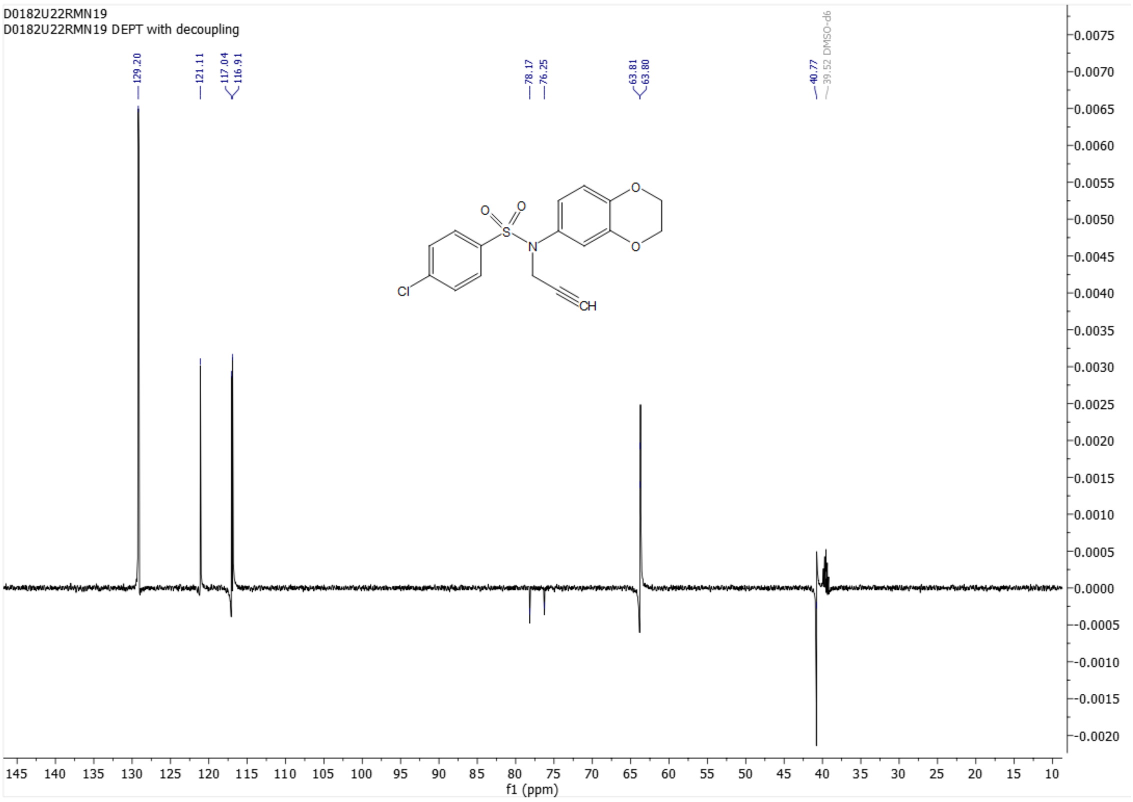

Figure S7. ^{13}C NMR DEPT 135 spectrum of compound **4b** in DMSO-d_6.

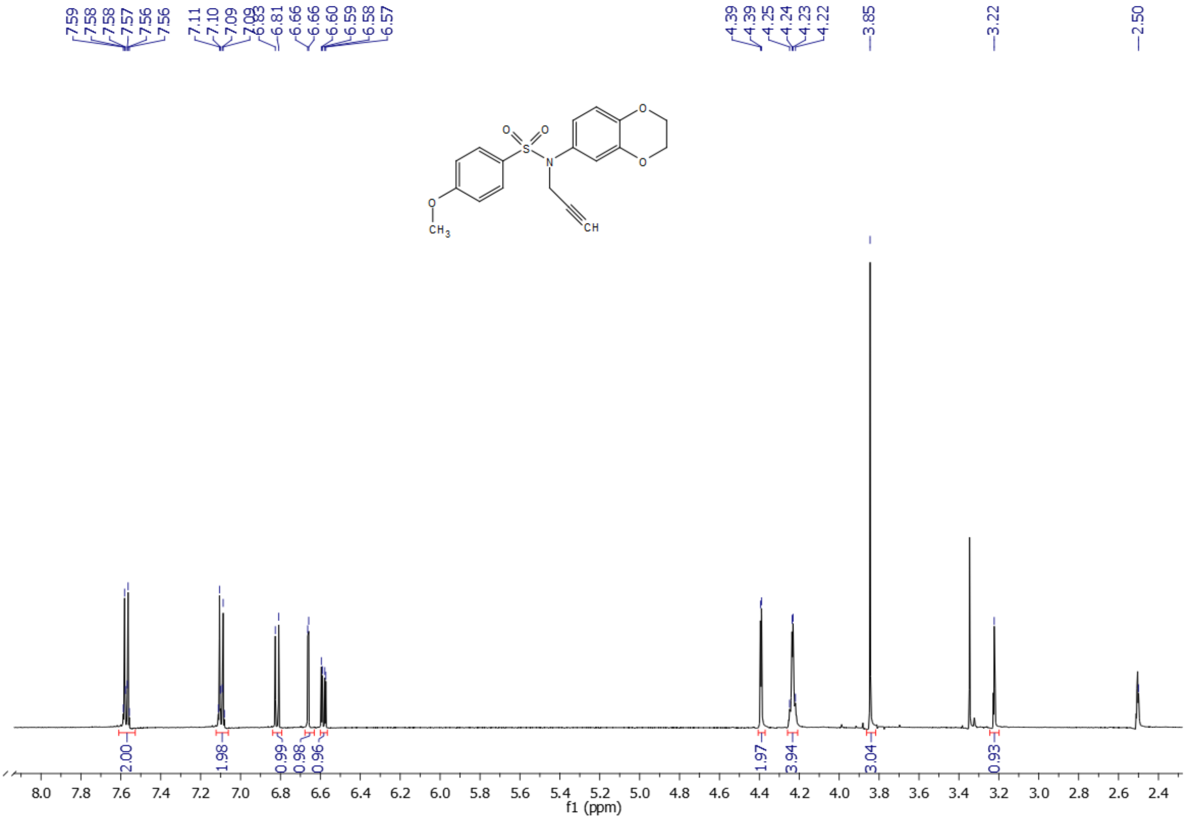

Figure S8. ^{1}H NMR spectrum of compound **4c** in DMSO-d_6.

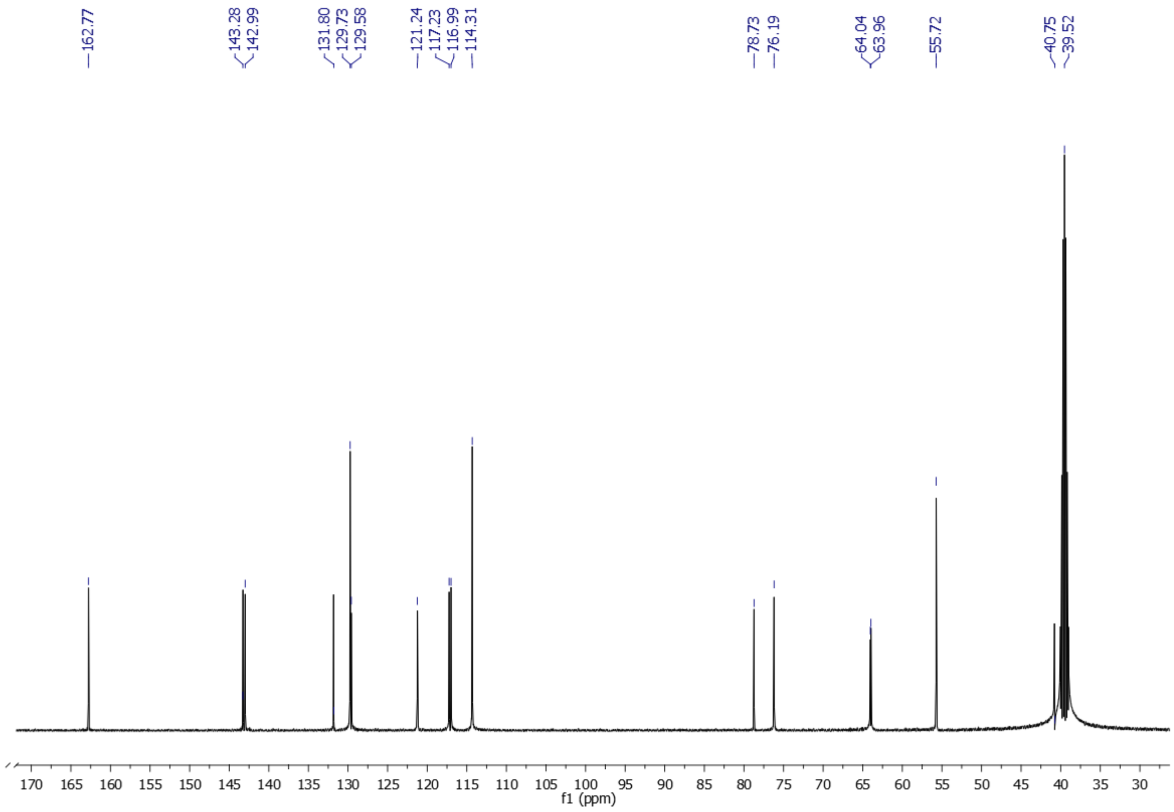

Figure S9. ^{13}C NMR spectrum of compound **4c** in DMSO-d_6.

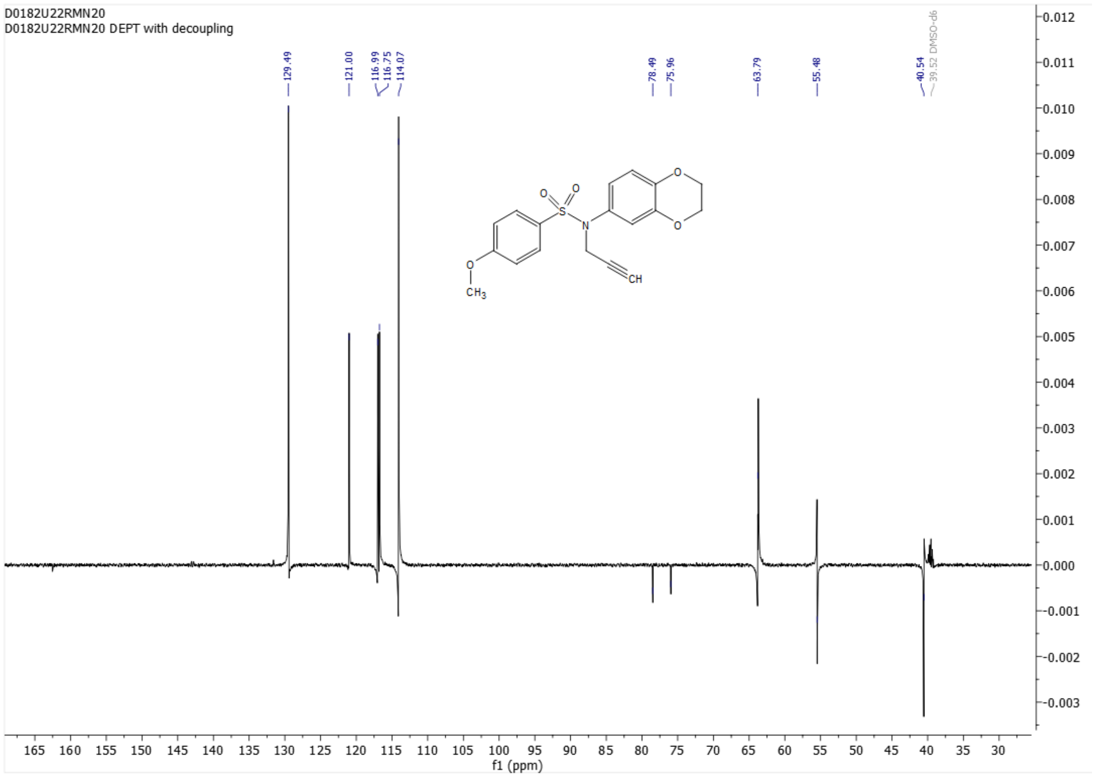

Figure S10. ^{13}C NMR DEPT 135 spectrum of compound **4c** in DMSO-d_6.

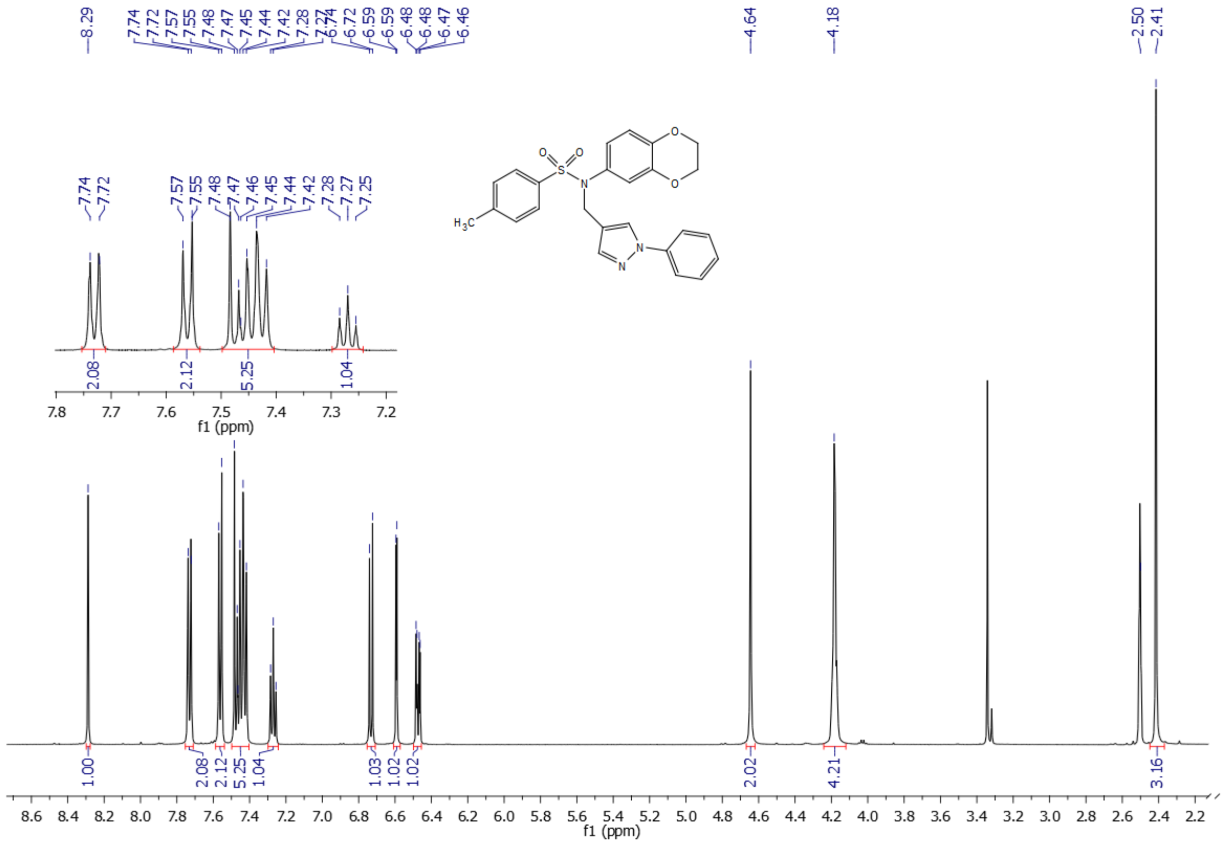

Figure S11. ^{1}H NMR spectrum of compound **5a** in DMSO-d_6.

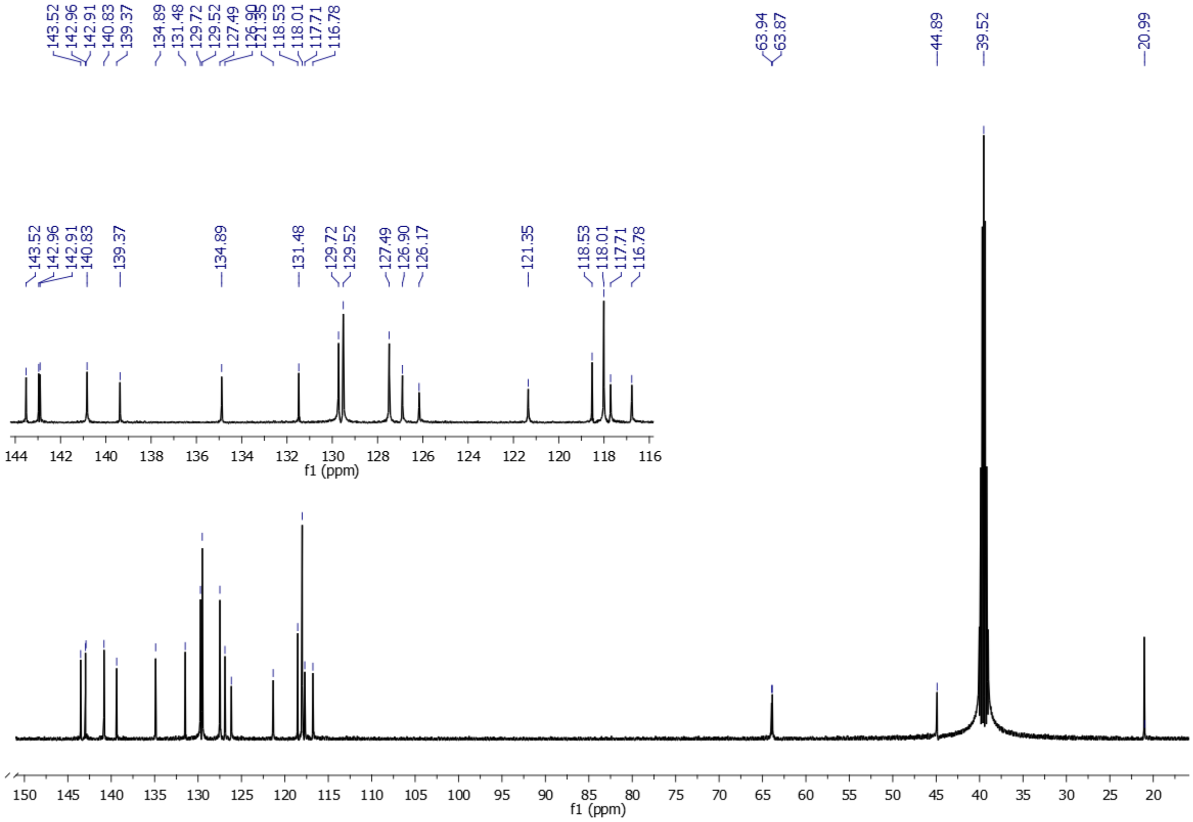

Figure S12. ^{13}C NMR spectrum of compound **5a** in DMSO-d_6.

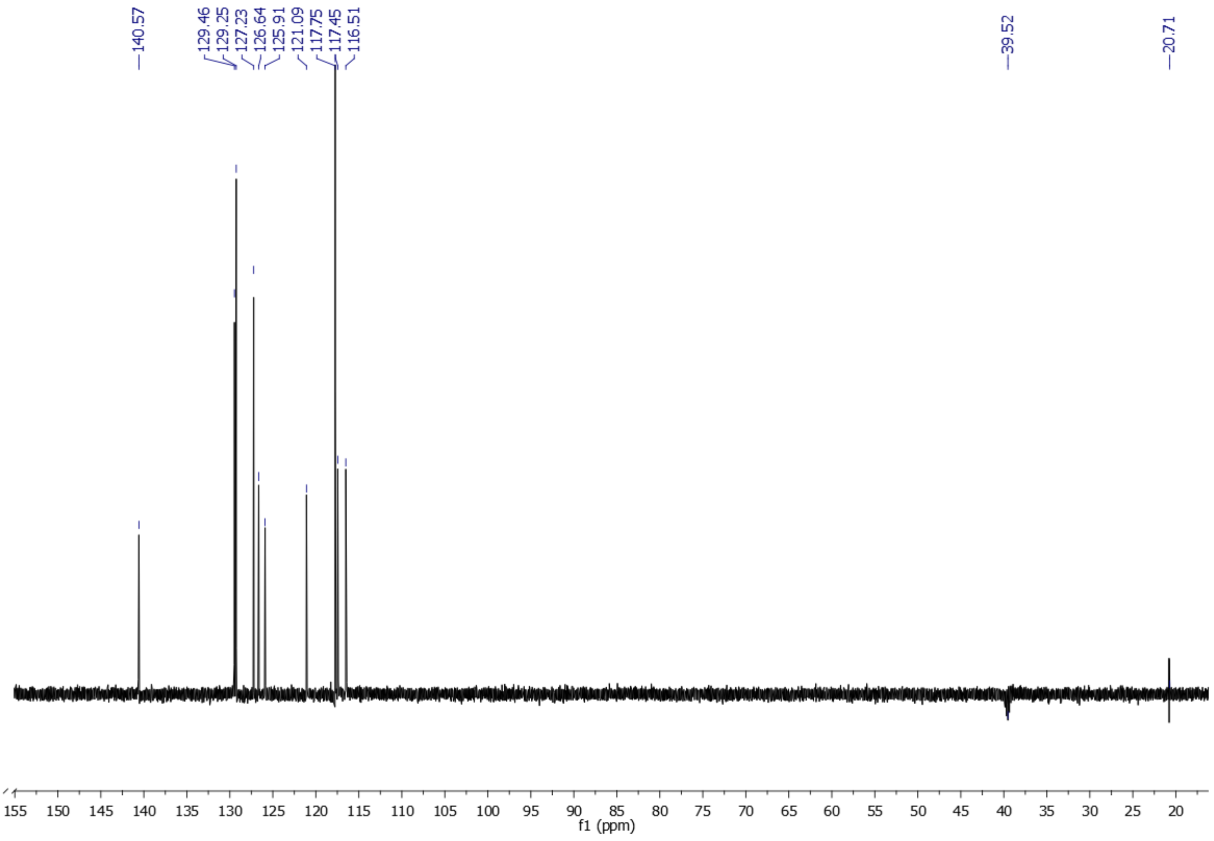

Figure S13. ^{13}C NMR DEPT 135 spectrum of compound **5a** in DMSO-*d*$_6$.

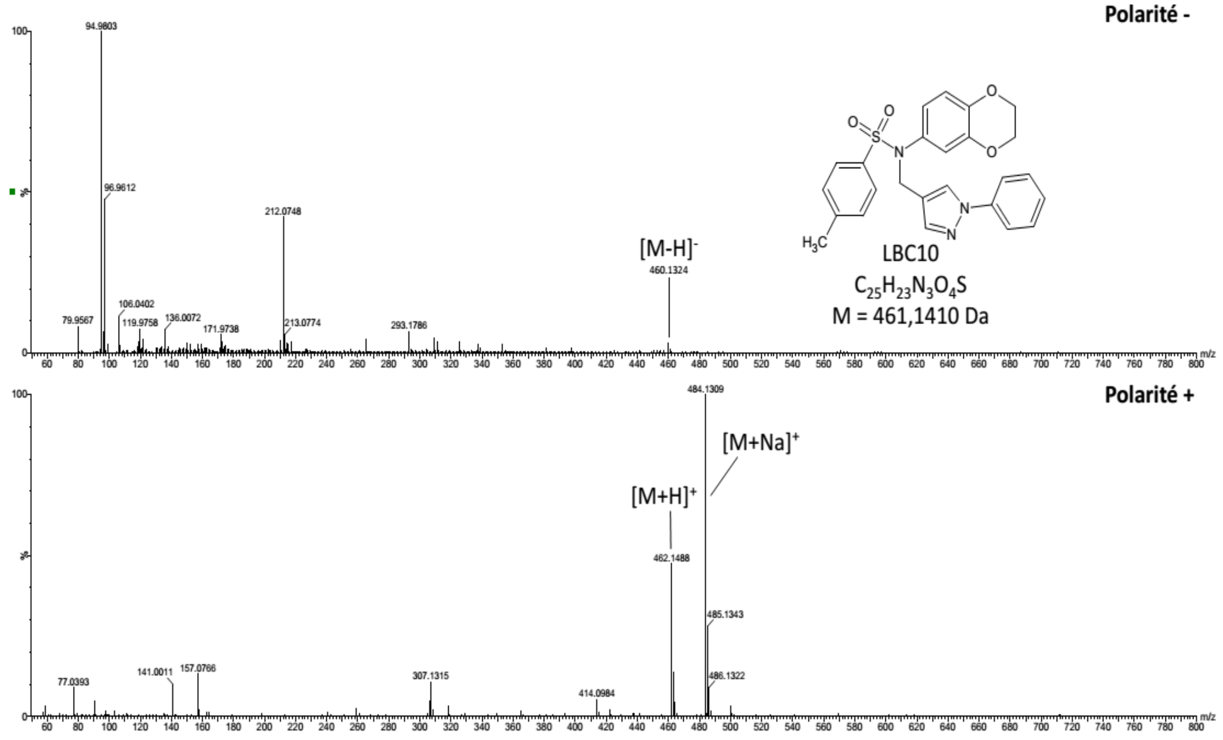

Figure S14. MS spectrum of compound **5a**

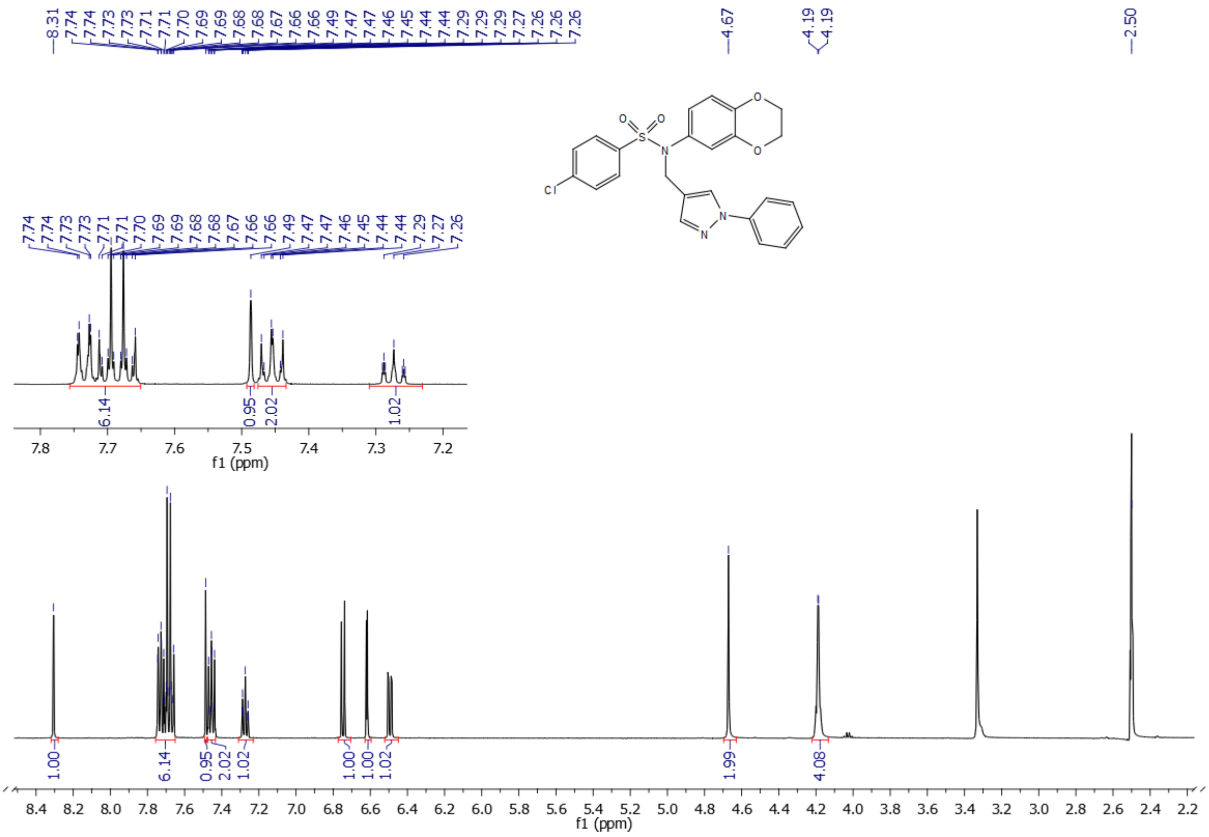

Figure S15. ^{1}H NMR spectrum of compound **5b** in DMSO-d_6.

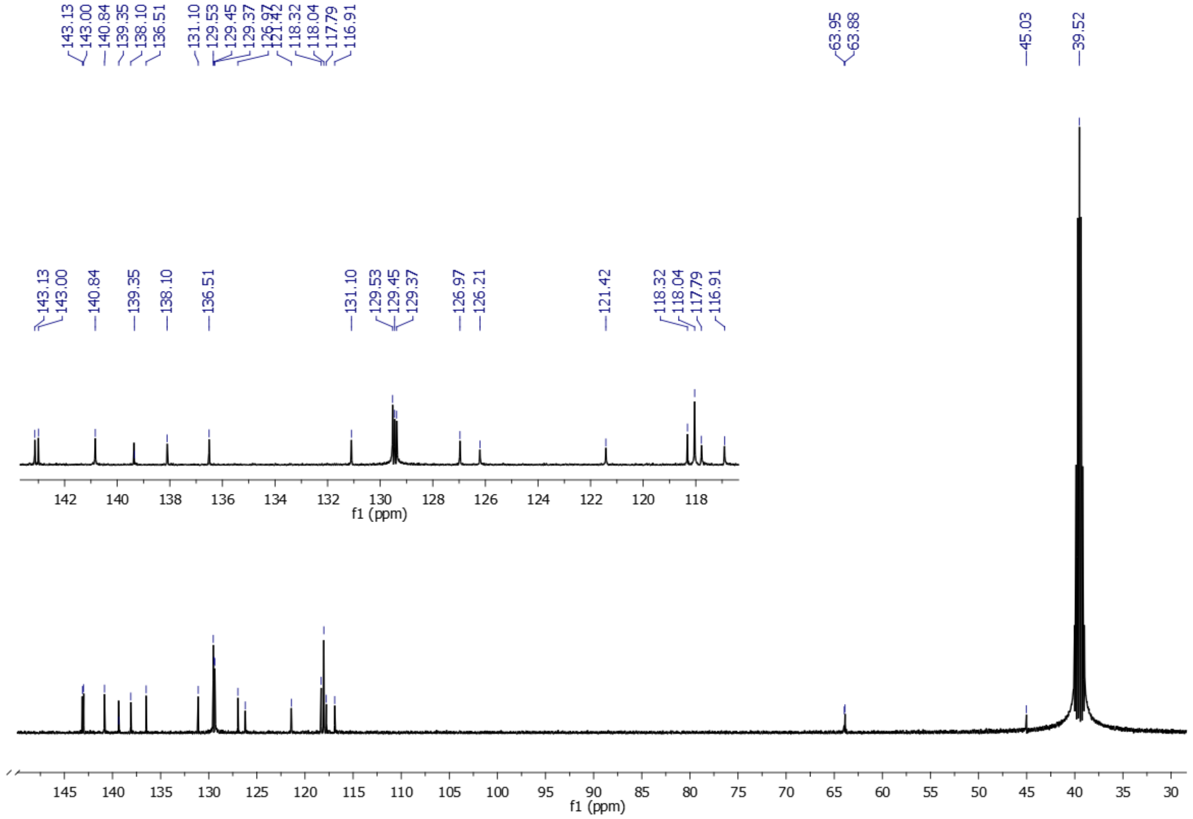

Figure S16. ^{13}C NMR spectrum of compound **5b** in DMSO-d_6.

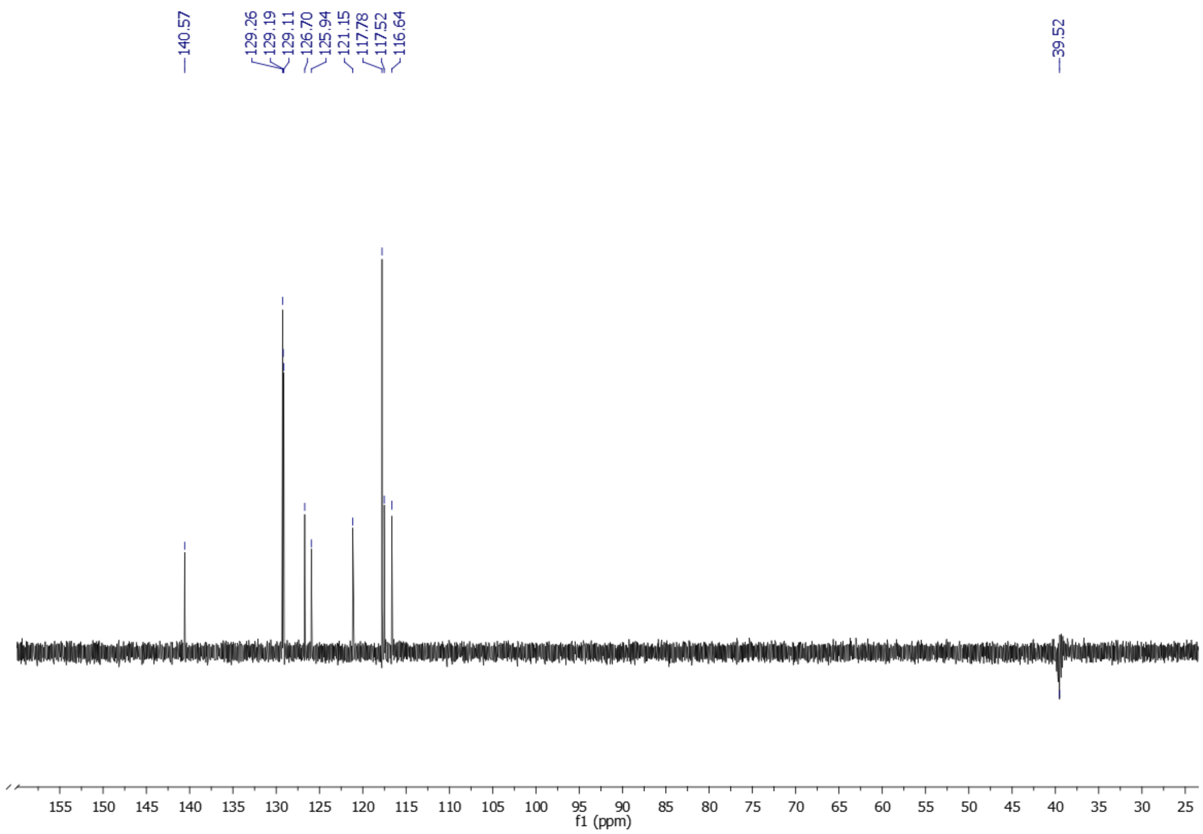

Figure S17. ^{13}C NMR DEPT 135 spectrum of compound **5b** in DMSO-d_6.

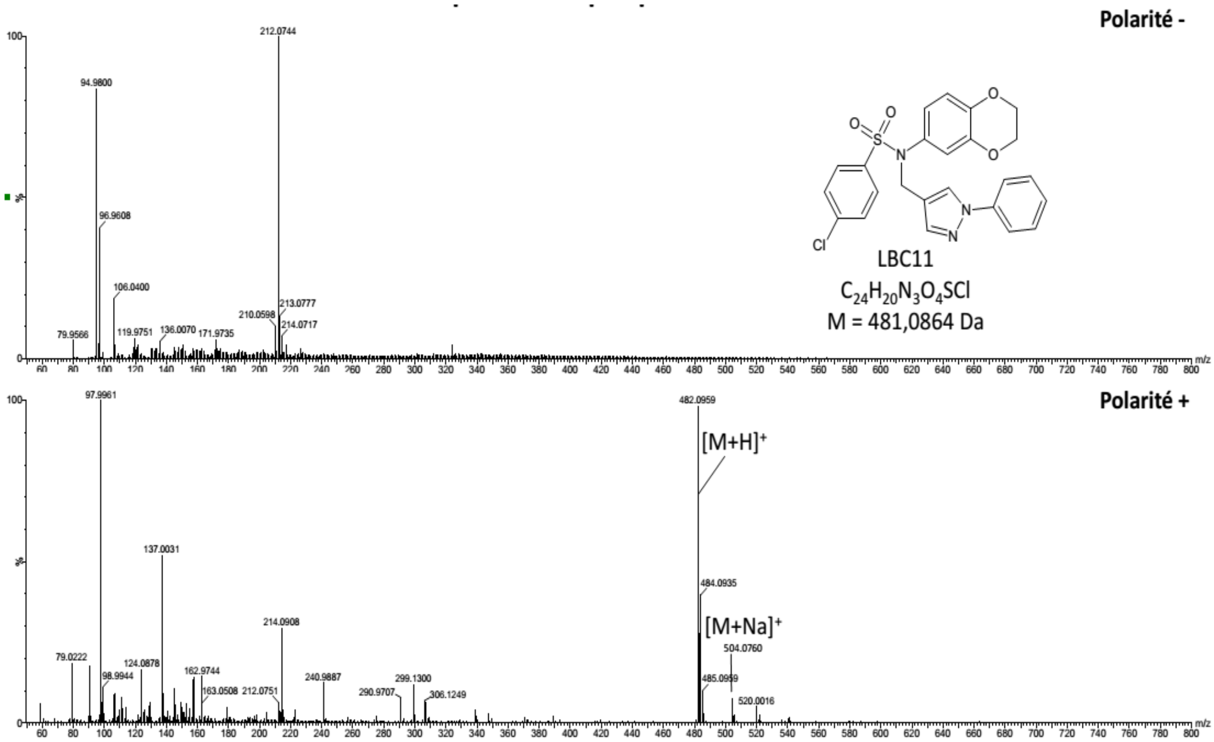

Figure S18. MS spectrum of compound **5b**

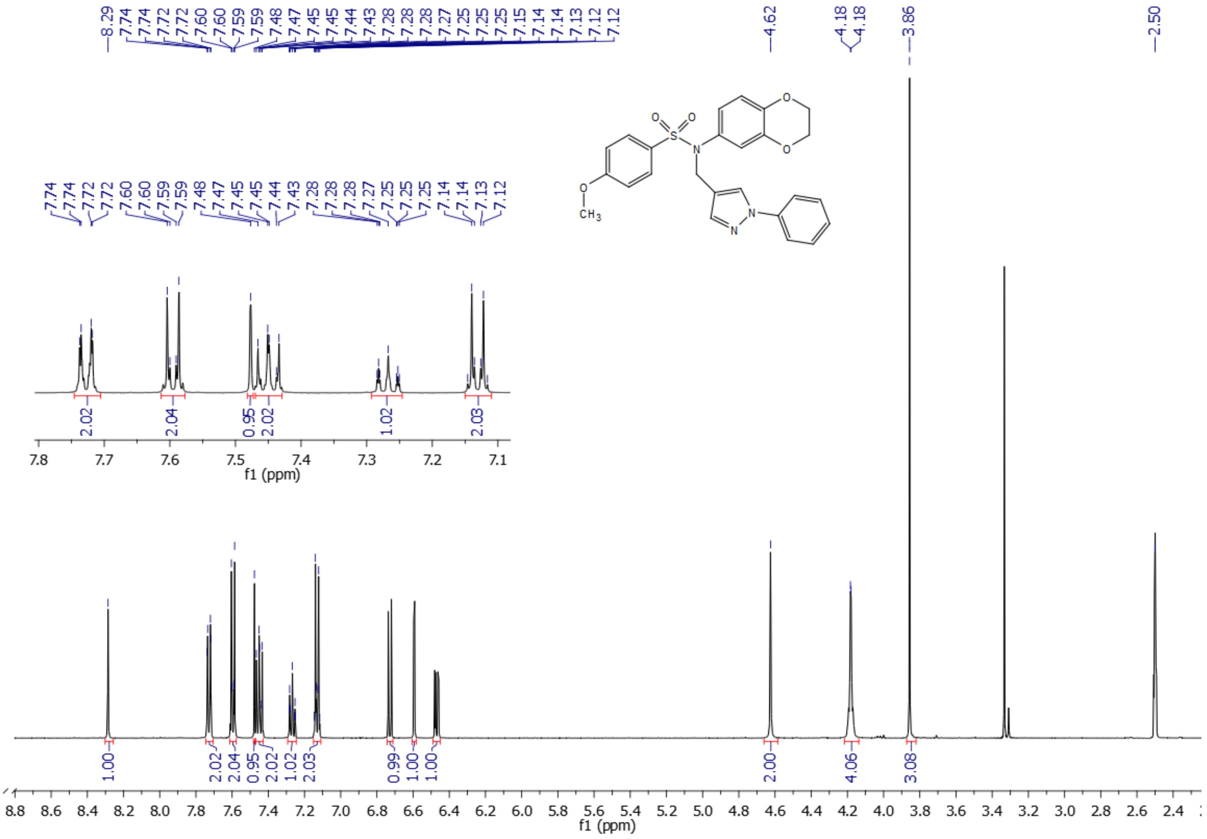

Figure S19. ^{1}H NMR spectrum of compound **5c** in DMSO-d_6.

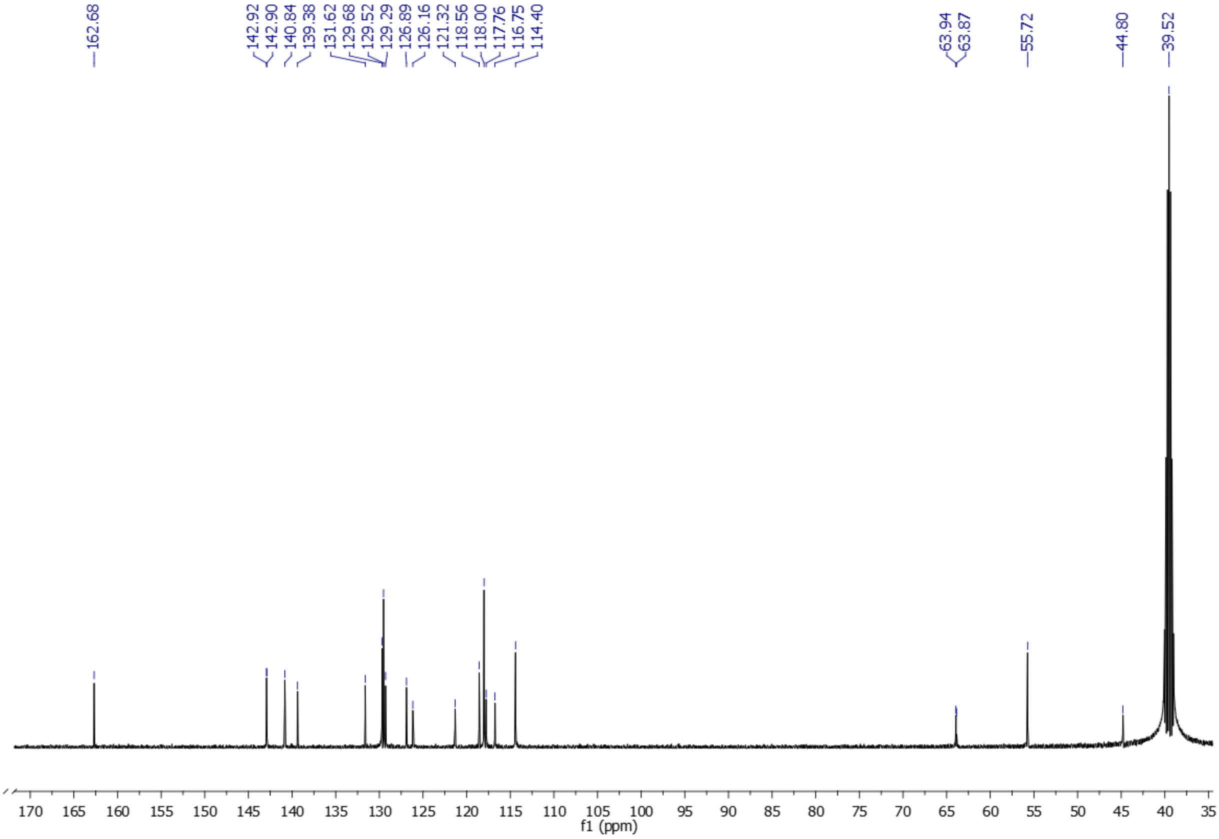

Figure S20. ^{13}C NMR spectrum of compound **5c** in DMSO-d_6.

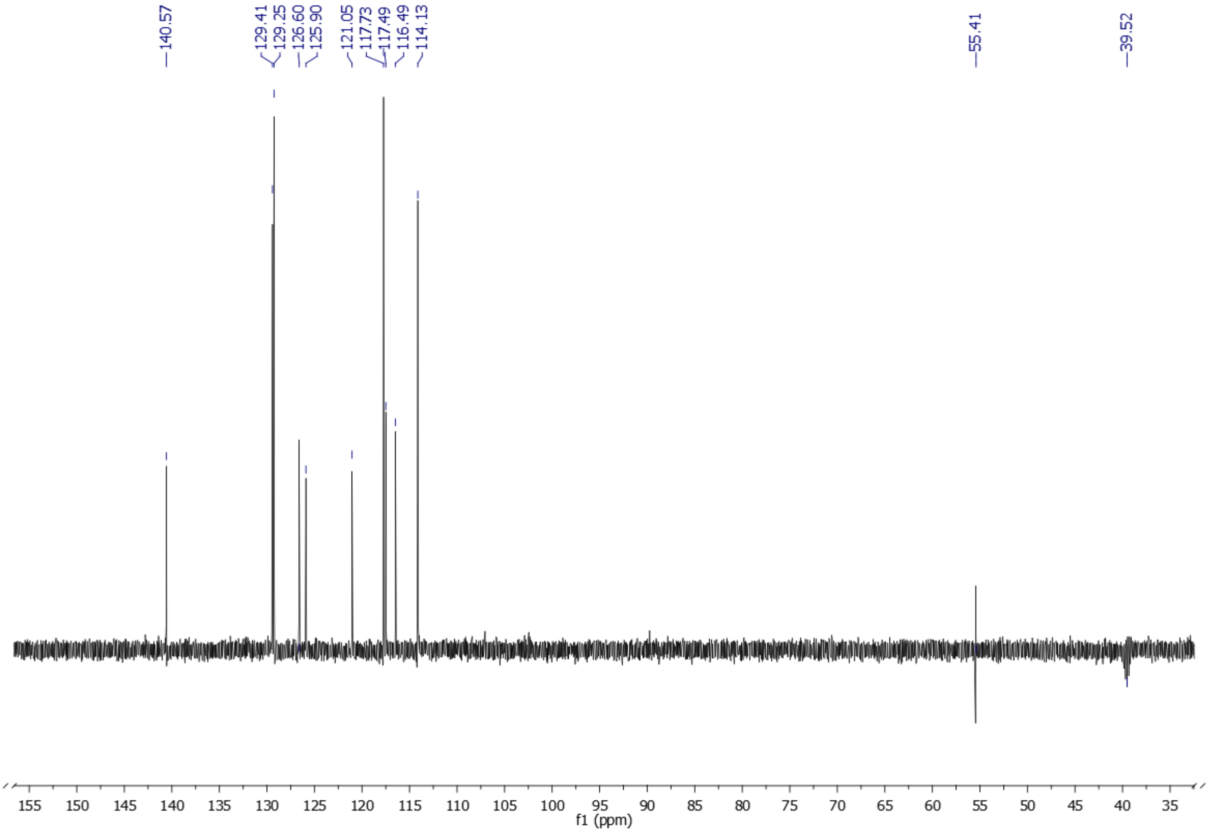

Figure S21. ^{13}C NMR DEPT 135 spectrum of compound **5c** in DMSO-*d6*.

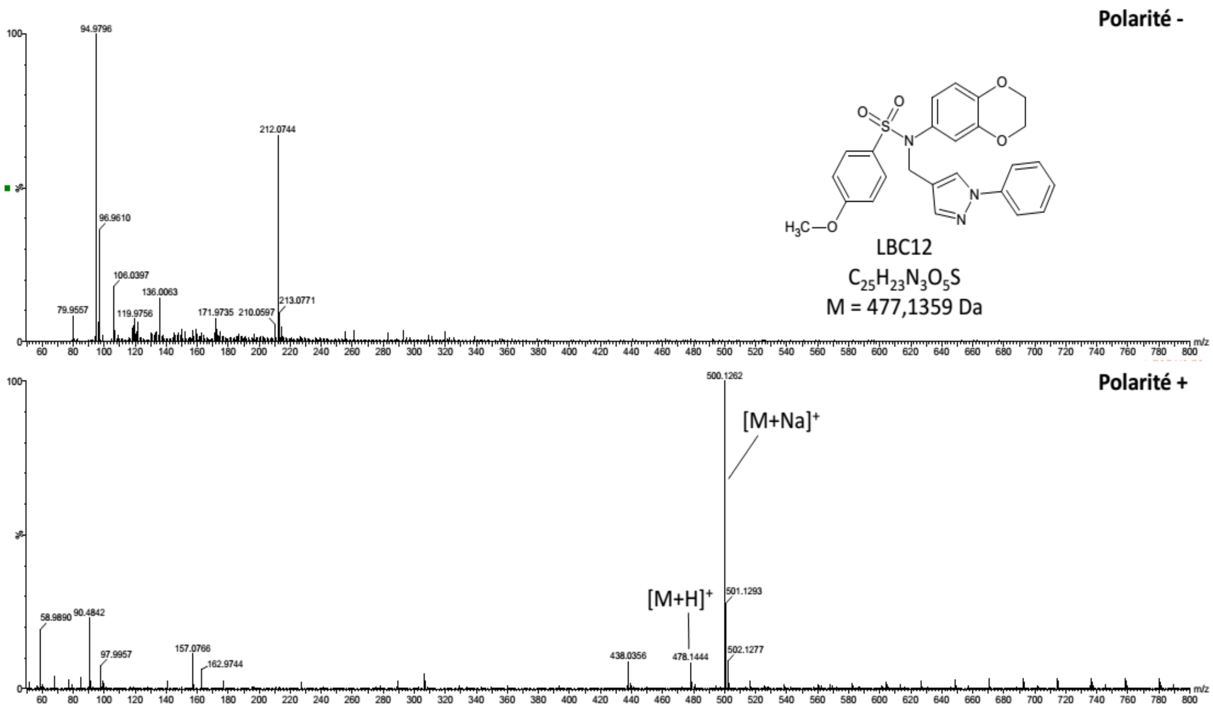

Figure S22. MS spectrum of compound **5c**

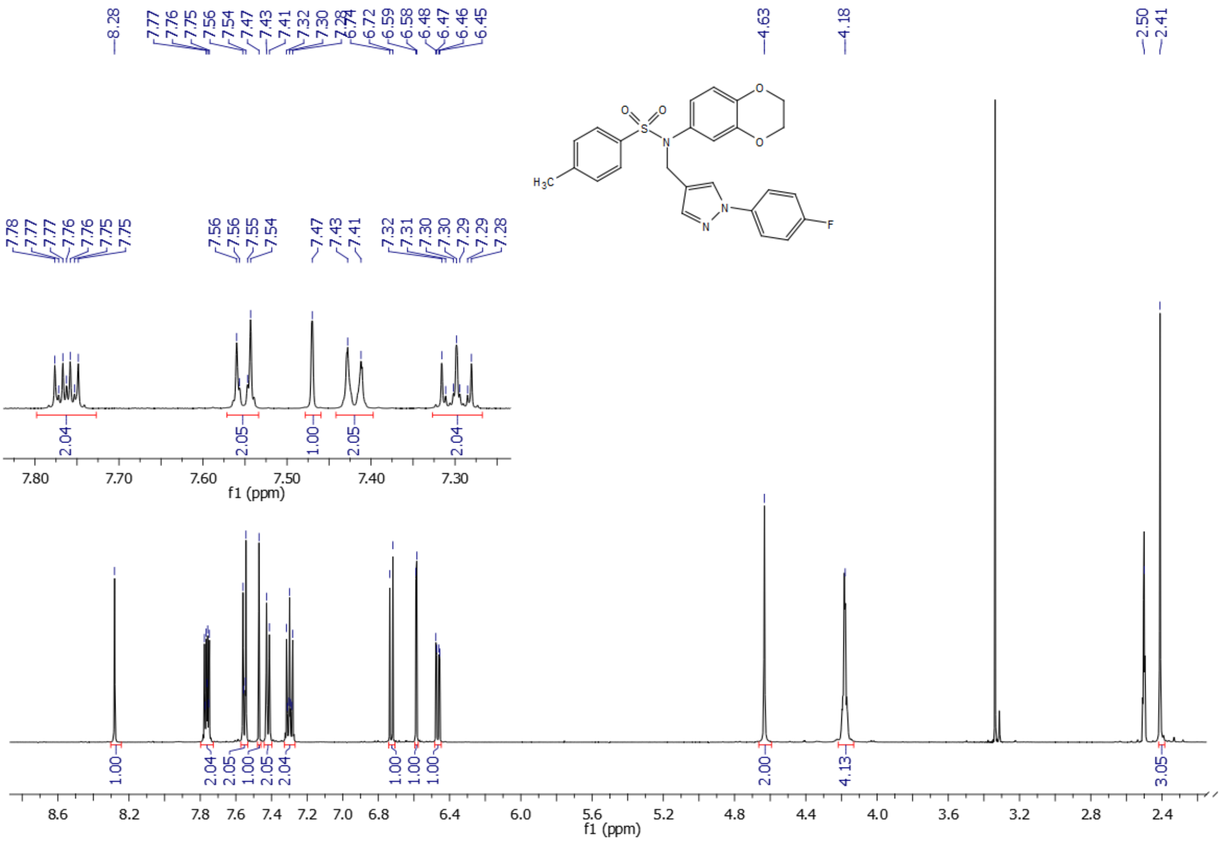

Figure S23. ^{1}H NMR spectrum of compound **5d** in DMSO-*d6*.

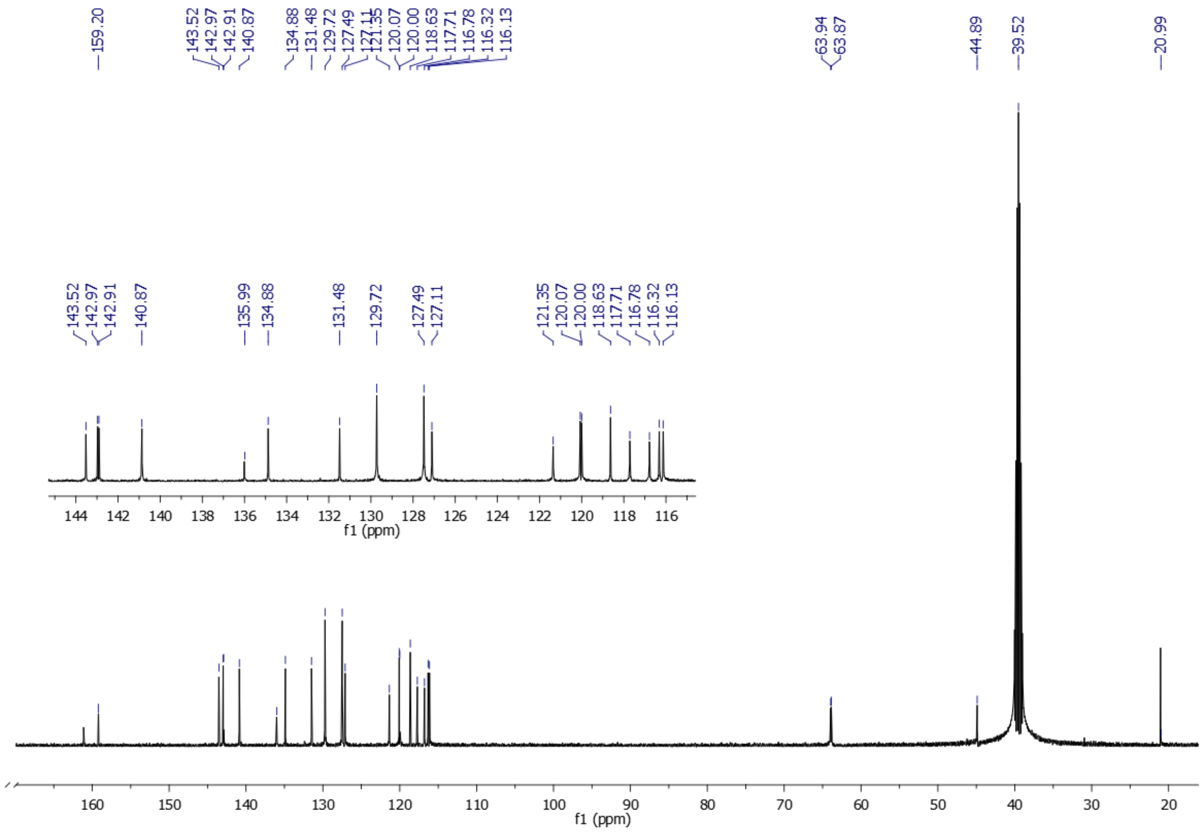

Figure S24. ^{13}C NMR spectrum of compound **5d** in DMSO-d_6.

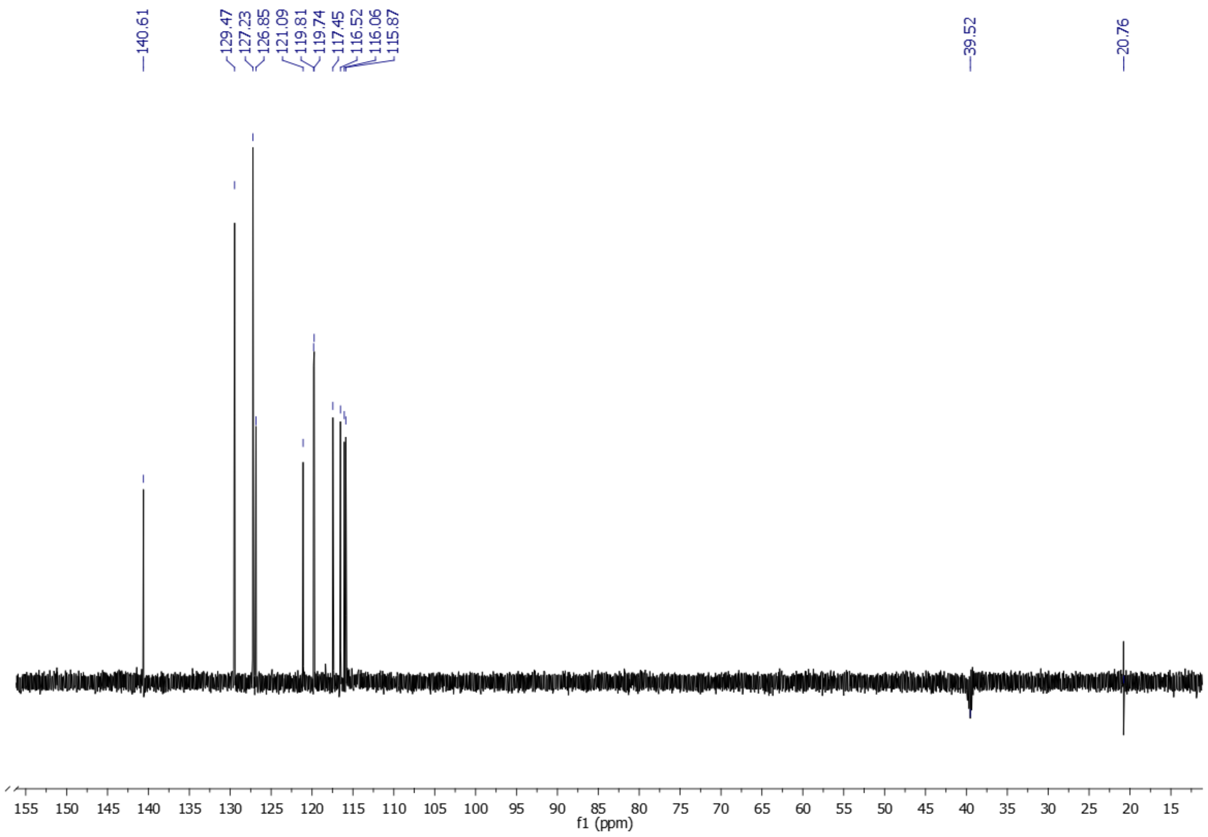

Figure S25. ^{13}C NMR DEPT 135 spectrum of compound **5d** in DMSO-d_6.

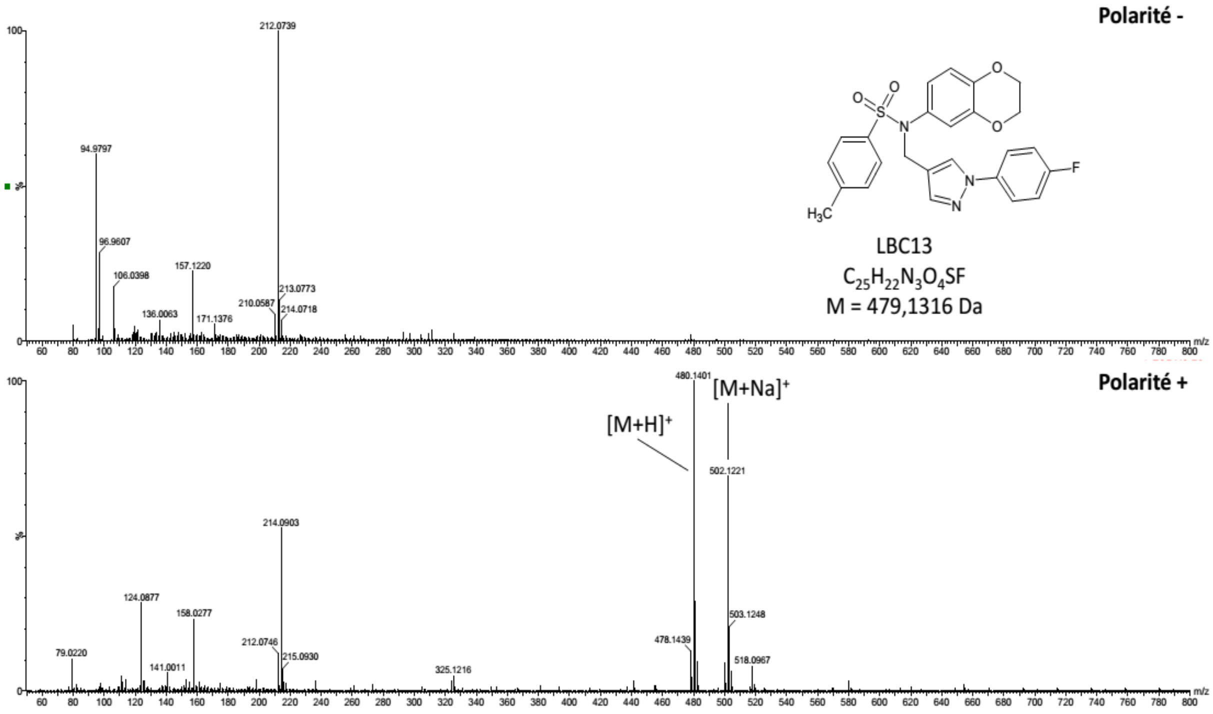

Figure S26. MS spectrum of compound **5d**

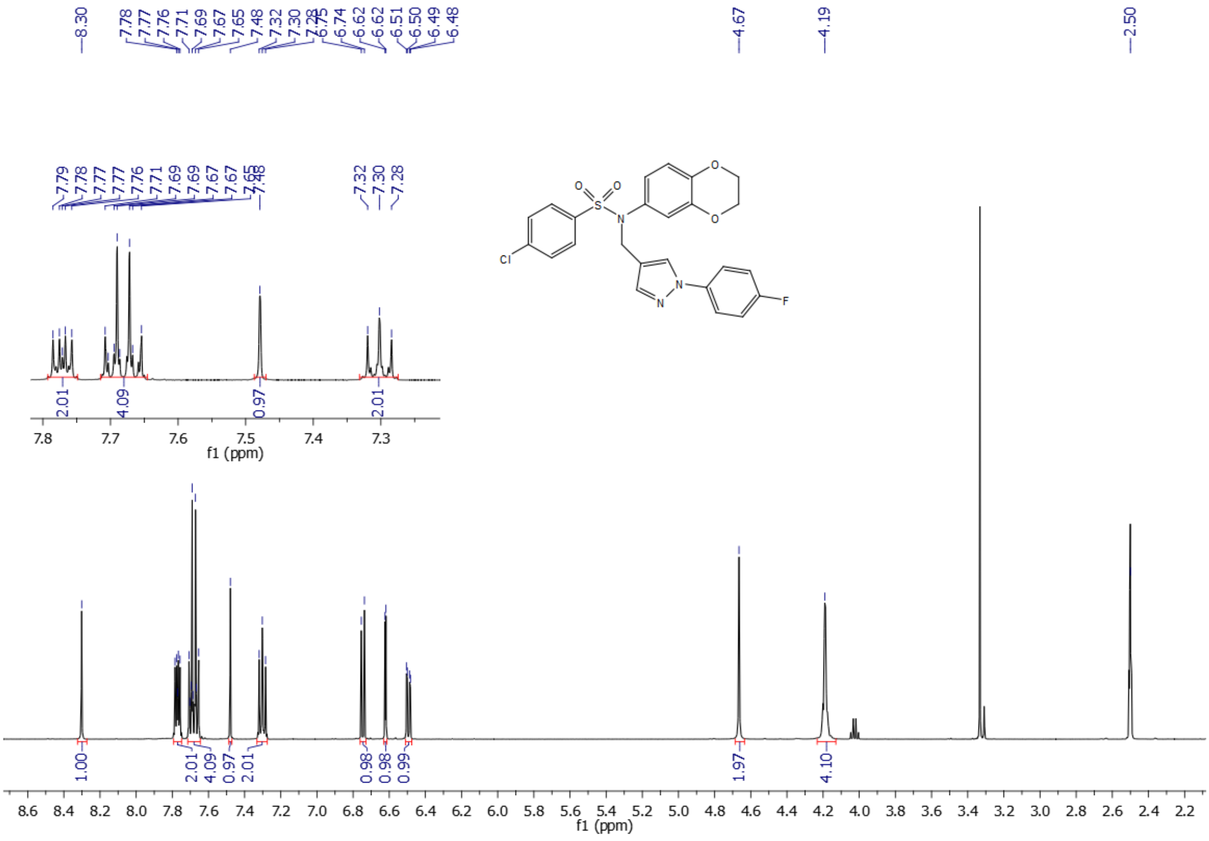

Figure S27. ^{1}H NMR spectrum of compound **5e** in DMSO-d_6.

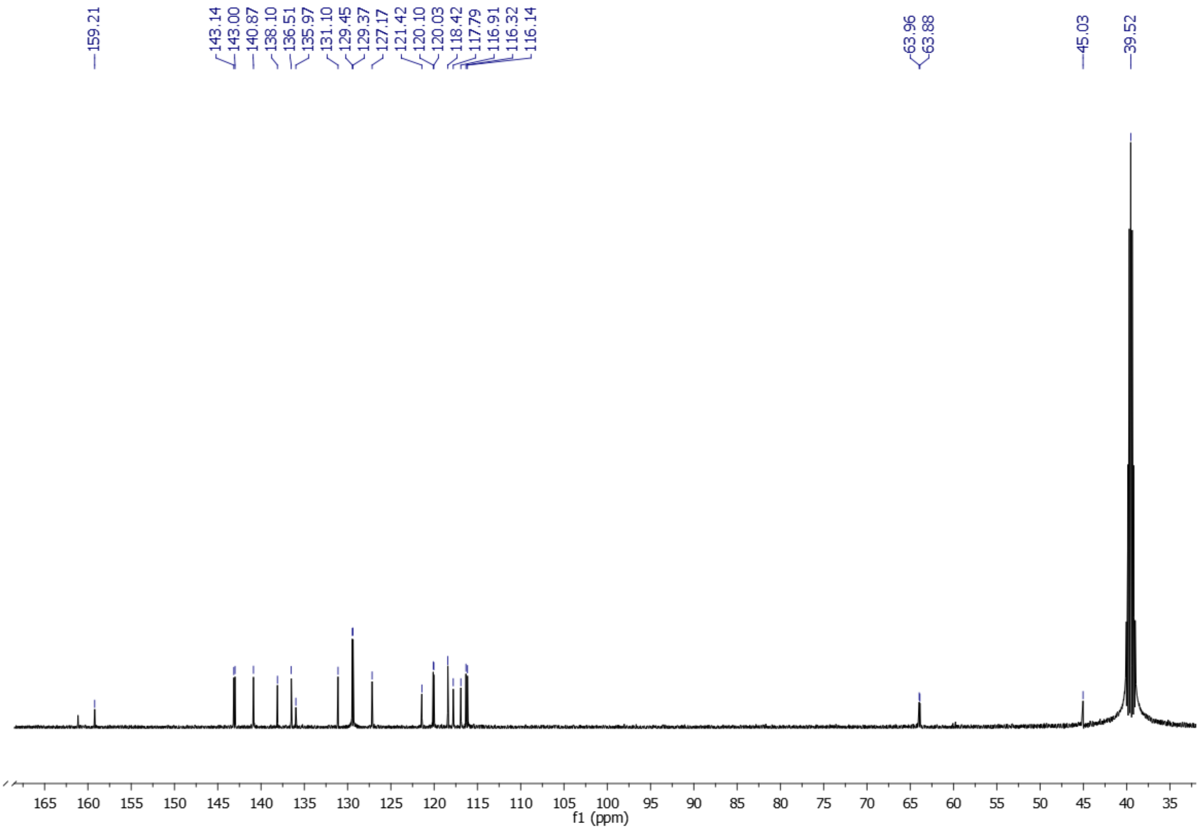

Figure S28. ^{13}C NMR spectrum of compound **5e** in DMSO-d_6.

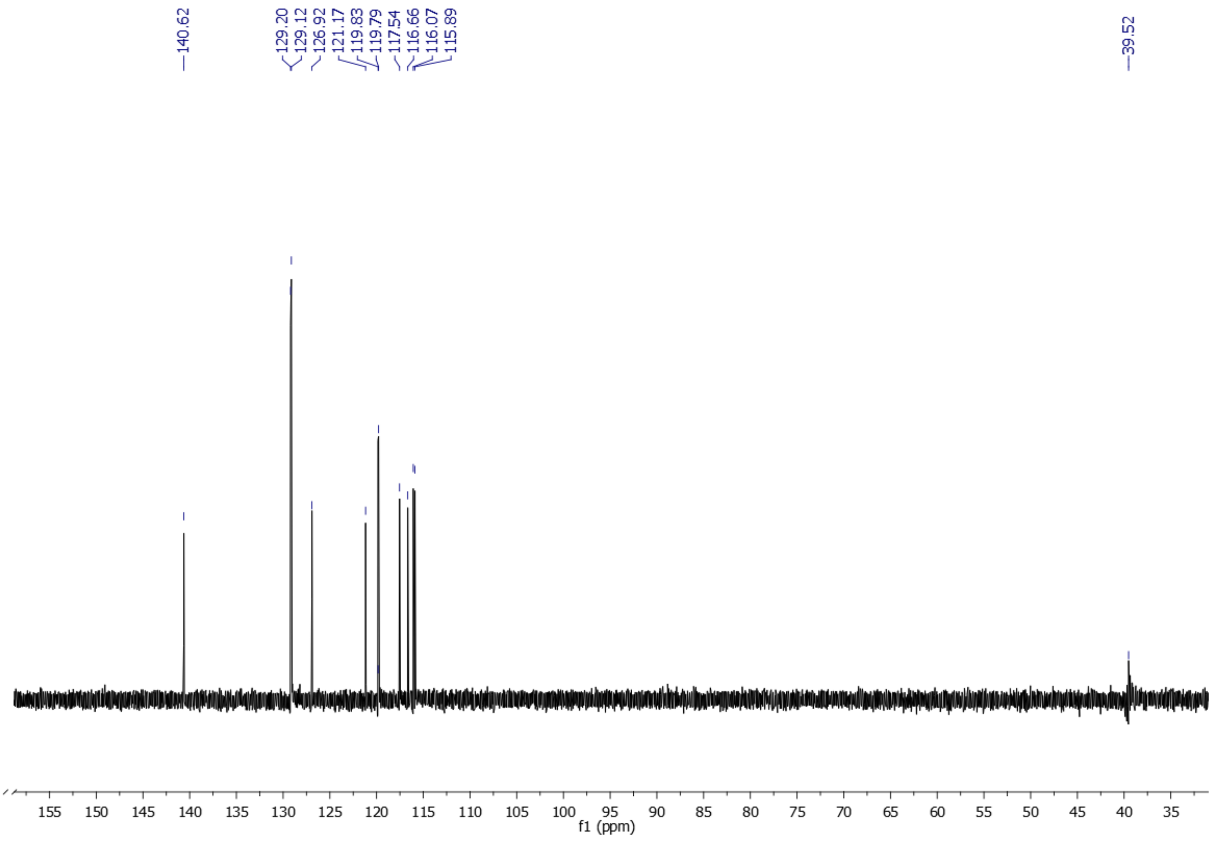

Figure S29. ^{13}C NMR DEPT 135 spectrum of compound **5e** in DMSO-d_6.

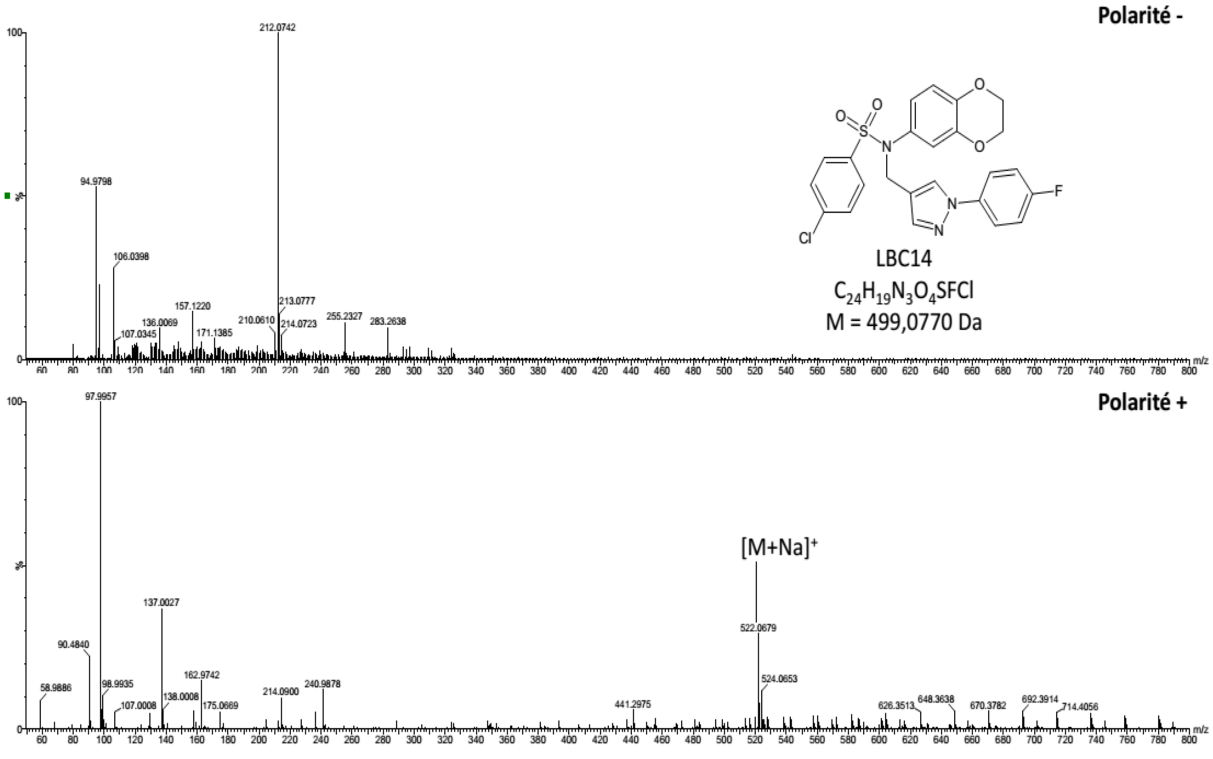

Figure S30. MS spectrum of compound **5e**

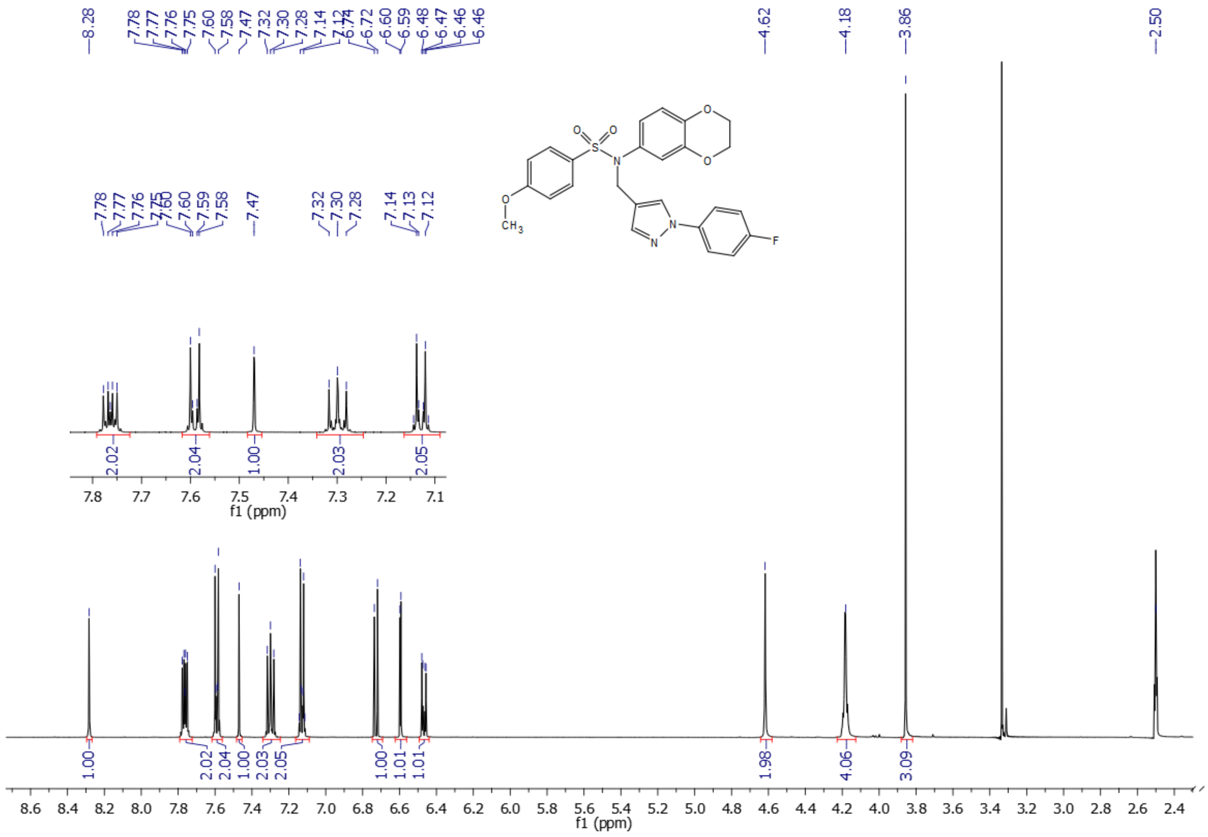

Figure S31. ^{1}H NMR spectrum of compound **5f** in DMSO-d_6.

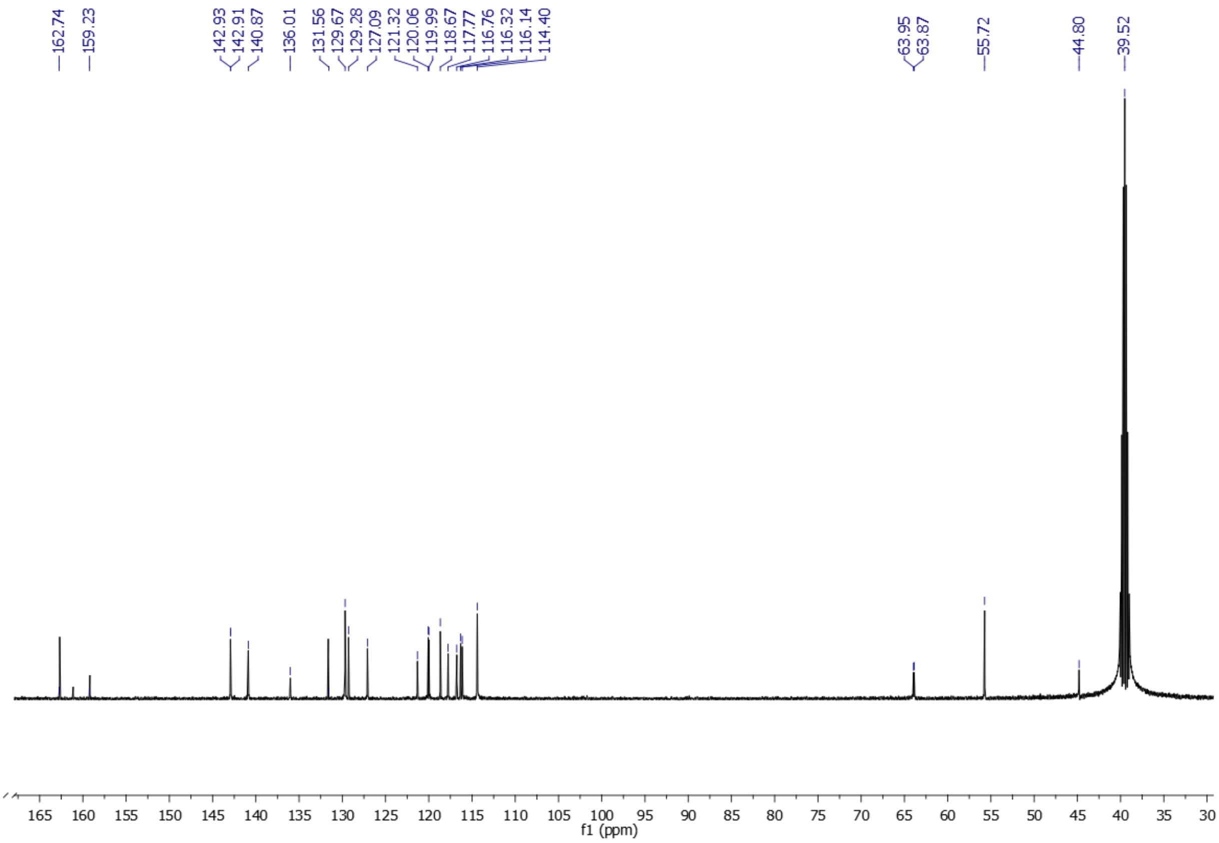

Figure S32. ^{13}C NMR spectrum of compound **5f** in DMSO-d_6.

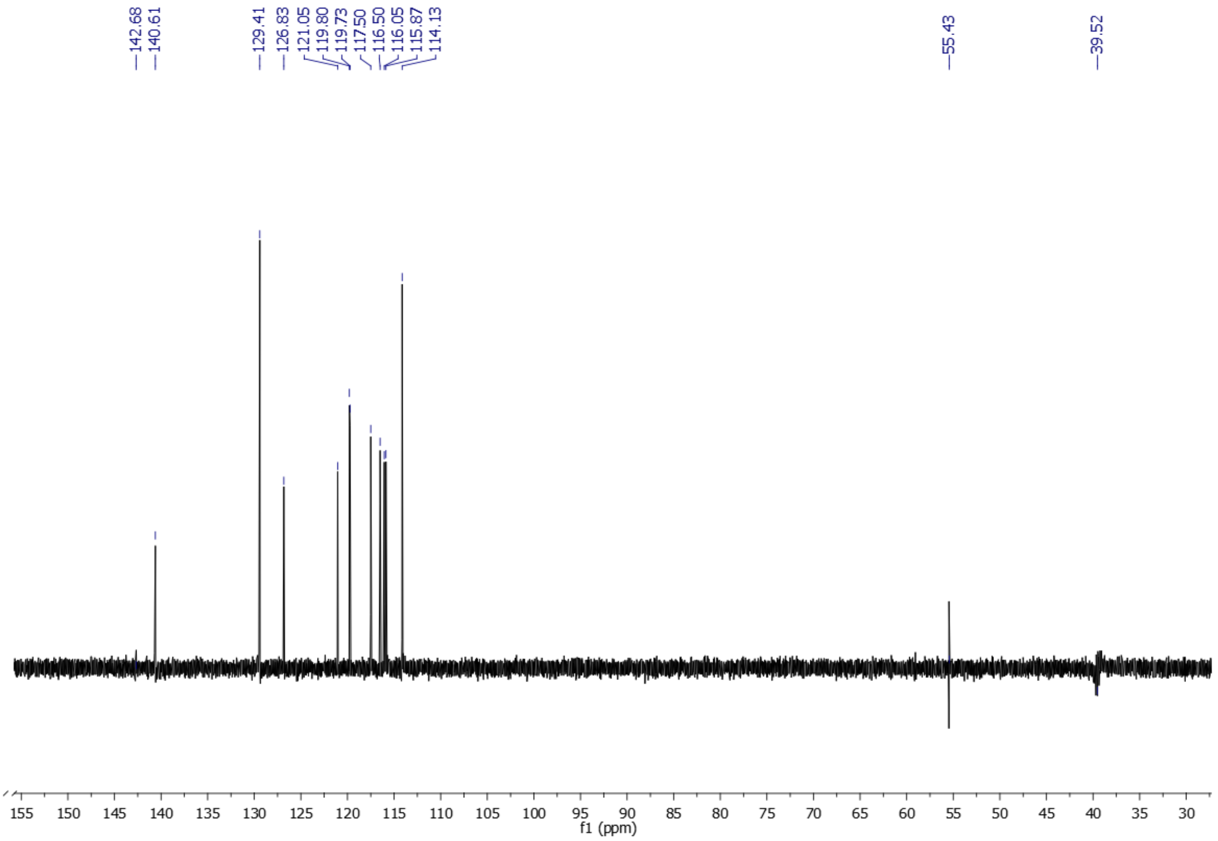

Figure S33. ^{13}C NMR DEPT 135 spectrum of compound **5f** in DMSO-d_6.

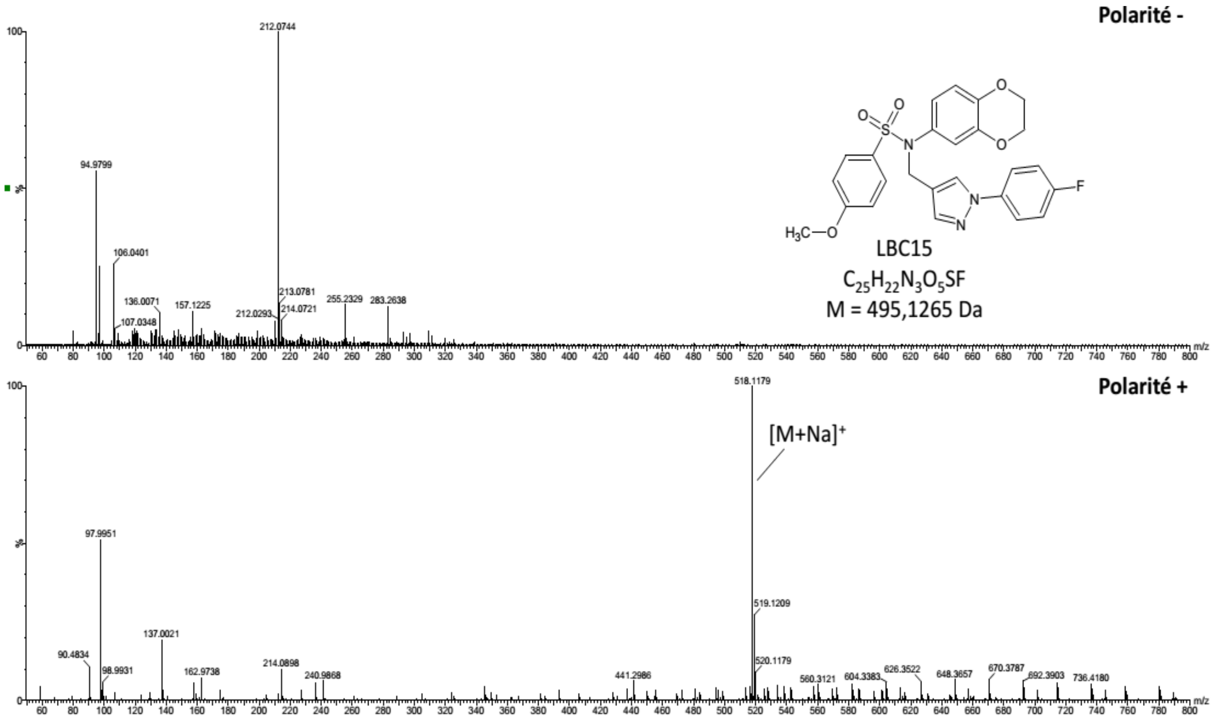

Figure S34. MS spectrum of compound **5f**
